# Efficacy and safety of left bundle branch pacing compared with left ventricular septal pacing: A systematic review and meta-analysis

**DOI:** 10.1016/j.hroo.2025.06.006

**Published:** 2025-08-13

**Authors:** Iwan Cahyo Santosa Putra, Raymond Pranata, Mohammad Iqbal, Giky Karwiky, Chaerul Achmad

**Affiliations:** Department of Cardiology and Vascular Medicine, Faculty of Medicine, Padjadjaran University, Bandung, Indonesia

**Keywords:** Left bundle branch area pacing, Left bundle branch pacing, Left ventricular septal pacing, Efficacy, Safety

## Abstract

**Background:**

Although numerous studies have compared the efficacy and safety of left bundle branch pacing (LBBP) and left ventricular (LV) septal pacing (LVSP), the results remain inconclusive.

**Objective:**

This meta-analysis aimed to systematically compare the efficacy and safety of LBBP with that of LVSP.

**Methods:**

A comprehensive literature search was conducted across PubMed, Europe PMC, and ScienceDirect to identify studies comparing procedural duration, complications, electrophysiological and echocardiographic parameters, and clinical outcomes between LBBP and LVSP.

**Results:**

A total of 22 cohort studies, involving 1360 LBBP and 1186 LVSP procedures, were included. The paced QRS duration (mean difference [MD] = −9.65 ms; 95% confidence interval [CI], −13.35 to −5.96; I^2^ = 84.9%; *P* < .001) and stimulus-to-LV activation time (MD = −14.62 ms; 95% CI, −16.99 to −12.24; I^2^ = 77.2%; *P* < .001) were significantly shorter in the LBBP group. In patients with reduced LV ejection fraction (LVEF) and wide QRS duration, the improvement in LVEF was significantly greater in the LBBP group (MD = 6.05%; 95% CI, 2.9–9.2; I^2^ = 51.3%; *P* < .001). In addition, the LBBP group demonstrated a significant reduction in the risk of all-cause mortality and/or heart failure hospitalization (risk ratio = 0.28; 95% CI, 0.17–0.48; I^2^ = 0%; *P* < .001). In patients with preserved LVEF and narrow QRS duration, postpacemaker implantation LVEF and LV end-diastolic diameter and the risk of heart failure hospitalization were comparable between the 2 groups. Furthermore, procedural duration, complications, and pacing parameters (sensing amplitude, capture threshold, and lead impedance) did not significantly differ between the groups.

**Conclusion:**

In patients with reduced LVEF and wide QRS duration, LBBP demonstrates superior efficacy compared with LVSP. In contrast, in patients with preserved LVEF and narrow QRS duration, the efficacy of LBBP and LVSP is similar. Both groups exhibit comparable safety profiles and procedural efficiency.

**Key Findings**

▪ In patients with reduced left ventricular (LV) ejection fraction (LVEF) and wide QRS duration, left bundle branch pacing (LBBP) has shown superior efficacy compared with LV septal pacing (LVSP) in maintaining intraventricular electrical synchrony. This led to more substantial improvements in LV systolic function and structural remodeling during follow-up. These benefits were further associated with a significant reduction in the risk of all-cause mortality and heart failure hospitalization (HFH).
▪ In patients with preserved LVEF and narrow QRS duration, although the LBBP group demonstrated better preservation of intraventricular synchrony, both groups exhibited similar efficacy in preserving LV systolic function and structural remodeling during follow-up, with comparable risks of HFH.
▪ Both LBBP and LVSP demonstrated comparable safety profiles and procedural efficiency.

## Introduction

The global use of pacemakers has risen significantly, with the highest implantation rates in North America (712 per million) and the lowest in South Asia and the Asia Pacific regions (52 per million).^1^, ^2^, ^3^ A meta-analysis by Somma et al^4^ found a 12% incidence of pacing-induced cardiomyopathy (PICM), highlighting the importance of selecting an optimal pacing site to ensure physiological conduction, reduce ventricular dyssynchrony, and minimize heart failure hospitalization (HFH) and mortality. In addition, factors such as higher procedural success rates, shorter procedural durations, fewer procedural complications, and lower lead capture threshold must also be considered.^5^ Thus, careful lead tip location selection is essential for improving long-term outcomes and procedural efficiency.

Right ventricular (RV) pacing, regardless of the site, can lead to left ventricular (LV) dysfunction, especially in patients with a high ventricular pacing burden.^6^ Early cardiac remodeling occurs within a month of RV pacing.^7^ Two meta-analyses showed that cardiac physiological pacing significantly improved LV systolic function, reduced mortality, and lowered HFHs compared with RV pacing.^8^^,^^9^ A meta-analysis by Wang et al^10^ comparing His-Purkinje system pacing, including His bundle pacing and left bundle branch (LBB) area pacing (LBBAP), with biventricular pacing found that His-Purkinje system pacing resulted in shorter QRS durations, higher LV ejection fraction (LVEF), reduced LV end-diastolic diameter (LVEDD), and a lower risk of HFH. In addition, Abdin et al’s^5^ meta-analysis reported that LBBAP outperformed His bundle pacing with higher R-wave amplitudes, lower capture thresholds, shorter procedural times, and higher success rates, while significantly reducing the risk of all-cause mortality and HFH.

The 2023 Heart Rhythm Society guidelines further classified LBBAP into 2 subtypes: LBB pacing (LBBP) and LV septal (LVS) pacing (LVSP).^11^ LBBP and LVSP were first introduced by Huang et al^12^ in 2017 and Mafi-Rad et al^13^ in 2016, respectively. In recent years, the use of both LBBP and LVSP has increased significantly among patients with bradycardia and heart failure.^14^, ^15^, ^16^, ^17^, ^18^, ^19^, ^20^, ^21^, ^22^, ^23^, ^24^, ^25^, ^26^, ^27^, ^28^, ^29^, ^30^, ^31^, ^32^, ^33^, ^34^, ^35^ The updated guidelines recommend LBBAP for patients requiring permanent pacing with a class IIa recommendation.^11^ However, specific recommendations for the selection of LBBP vs LVSP are yet to be established. Despite numerous cohort studies comparing their safety and efficacy, the results remain equivocal.^14^, ^15^, ^16^, ^17^, ^18^, ^19^, ^20^, ^21^, ^22^, ^23^, ^24^, ^25^, ^26^, ^27^, ^28^, ^29^, ^30^, ^31^, ^32^, ^33^, ^34^, ^35^ This meta-analysis aimed to compare procedural duration, complications, electrophysiological and echocardiographic parameters, and clinical outcomes between LBBP and LVSP to determine the most effective and safe pacing strategy.

## Methods

### Protocol and registration

This meta-analysis was registered in the PROSPERO (International Prospective Register of Systematic Reviews) database with the registration number CRD42025645190, and it adhered to the Preferred Reporting Items for Systematic Reviews and Meta-Analyses guidelines.

### Literature search strategy

Two independent reviewers conducted a comprehensive search of MEDLINE (via PubMed), Europe PMC, and ScienceDirect databases from their inception to February 2025, using the search terms (“left bundle branch pacing”) AND (“left ventricular septal pacing”). We used a streamlined set of keywords to refine the scope of the inquiry efficiently while maximizing the retrieval of relevant articles. Furthermore, the search terms were tailored to the specific requirements of each database. Full-text articles were rigorously assessed by both authors (I.C.S.P. and R.P.), and any discrepancies regarding the inclusion of studies were resolved through discussion.

### Eligibility criteria and outcomes of interest

This meta-analysis incorporated both randomized controlled trials and cohort studies (prospective or retrospective) that encompassed multiple variables. First, the study should include patients (age ≥18 years) requiring permanent pacemaker implantation. The indications for pacemaker implantation encompass sinus node dysfunction, atrioventricular block, atrial fibrillation (AF) with bradycardia, or cardiac resynchronization therapy (CRT) candidates with heart failure and/or bundle branch block. Second, the study should provide a comparative analysis of LBBP and LVSP. Third, eligible studies were required to report at least 1 of the following outcomes: procedural duration (lead implantation time, implant fluoroscopy time, and procedural time), periprocedural complications (interventricular septal perforation and lead dislodgement), electrophysiological parameters (pacing sensing amplitude, pacing capture threshold, pacing impedance, ventricular pacing burden, paced QRS duration, stimulus-to-LV activation time [s-LVAT], V1 R-wave peak time [RWPT], and V1–V6 interpeak interval), echocardiographic parameters (LVEF and LVEDD), and clinical outcomes (all-cause mortality and HFH).

We excluded review articles, editorials, commentaries, case reports/series, meta-analyses, conference abstracts, and studies published in languages other than English. A third investigator (M.I.) served as the adjudicator, and any discrepancies were resolved through discussion.

### LBBP and LVSP procedure and pacing criteria

The LBBAP procedure was conducted via transvenous access. Angiographic imaging was performed in multiple views, with intermittent small injections of contrast medium to guide lead placement and minimize the risk of septal perforation. Low-output pacing (<1 V, 0.5 ms) was used to allow real-time monitoring of both the electrocardiogram and intracardiac electrogram. The pacing lead was inserted perpendicularly into the interventricular septum (positioned 1.0–1.5 cm below the His bundle site) using clockwise rotation, moving approximately 6–8 mm from the right to the left side until it reached the endocardial LVS, as indicated by a right bundle branch block pattern in lead V1. Subsequently, LBB capture was identified to confirm successful installation of the LBBP. In contrast, LVSP presents with a right bundle branch block pattern in lead V1, but without meeting the criteria for LBB capture.^11^^,^^13^^,^^36^

The definition of LBB capture varies across studies, but typically includes several key electrophysiological parameters: (1) a ≥10 ms reduction in LV activation time (LVAT) with increased pacing output; (2) a sudden increase in the V6 RWPT of >15 ms at reduced pacing output; (3) an abrupt shortening of the interval from s-LVAT, which equals to the interval from LBB potential to LVAT (LBB po-LVAT) (±10 ms or ±5 ms) and remains stable across pacing outputs; (4) an s-LVAT of <80 ms or <76 ms; (5) an s-LVAT of <75 ms for narrow QRS complexes and <80 ms for LBB block (LBBB) or interventricular conduction delay (IVCD); (6) transition from nonspecific to specific LBB capture as pacing output decreases without prolongation of RWPT or s-LVAT; and (7) a V6–V1 interpeak interval of >33 ms or >40 ms or >44 ms.^14^, ^15^, ^16^, ^17^, ^18^, ^19^, ^20^, ^21^, ^22^, ^23^, ^24^, ^25^, ^26^, ^27^, ^28^, ^29^, ^30^, ^31^^,^^33^, ^34^, ^35^

### Study selection and data collection

The initial search and removal of duplicate records were conducted independently by 2 investigators (I.C.S.P. and R.P.). Titles and abstracts were screened for eligibility, and full-text articles of potentially eligible studies were assessed according to predefined inclusion and exclusion criteria. Data extraction from the eligible studies was performed using a standardized template that included the following variables: first author’s name, publication year, country of origin, total number of participants, indications for pacing, subgroups, pacing method (LBBP or LVSP), age, sex, baseline LVEF, baseline QRS duration, exclusion criteria, mean follow-up duration, and outcomes.

### Risk of bias assessment

The risk of bias for each study was independently assessed by 2 authors (I.C.S.P. and R.P.) using the Newcastle-Ottawa scale (NOS). Studies with a total score of 7 or higher were deemed to have a low risk of bias. In contrast, studies scoring 6 or lower were considered to have a substantial risk of bias and were excluded from the selection process. Any disagreements regarding quality ratings were resolved through discussion with a third reviewer (M.I.).

### Data analysis

Statistical analyses were conducted using Stata software (version 17.0; StataCorp, College Station, TX). For categorical variables, the effect size for the meta-analysis was expressed as risk ratios with 95% confidence intervals. For continuous variables, the mean difference was used as the measure of effect. Values reported as medians with interquartile ranges were converted to means ± standard deviations for analysis.^37^ The restricted maximum likelihood method, coupled with random-effects models, was used to estimate the overall effect size, irrespective of the heterogeneity status. All statistical tests were 2 tailed, with a significance level set at a *P* value of <.05.^38^ Interstudy heterogeneity was assessed using the I^2^ statistic, where values exceeding 50% or *P* values of <.10 indicated significant heterogeneity.^39^ A sensitivity analysis was performed using the leave-one-out approach to identify potential sources of interstudy heterogeneity.^40^ The subgroup analysis was conducted to evaluate the pooled effect size within each subgroup and to identify potential sources of heterogeneity.^41^ The subgroup analysis was conducted based on LVEF, QRS duration, and LVEDD. LVEF was considered preserved if it was 50% or greater and reduced if it was less than 50%.^42^ QRS duration was classified as wide if it exceeded 120 ms and narrow if it was 120 ms or less.^43^ LVEDD was categorized as large if it was 55 mm or greater and small if it was less than 55 mm.^44^ Publication bias was evaluated qualitatively through Begg’s funnel plot and quantitatively via Egger’s test.^38^

## Results

### Baseline characteristics

**Figure 1:**
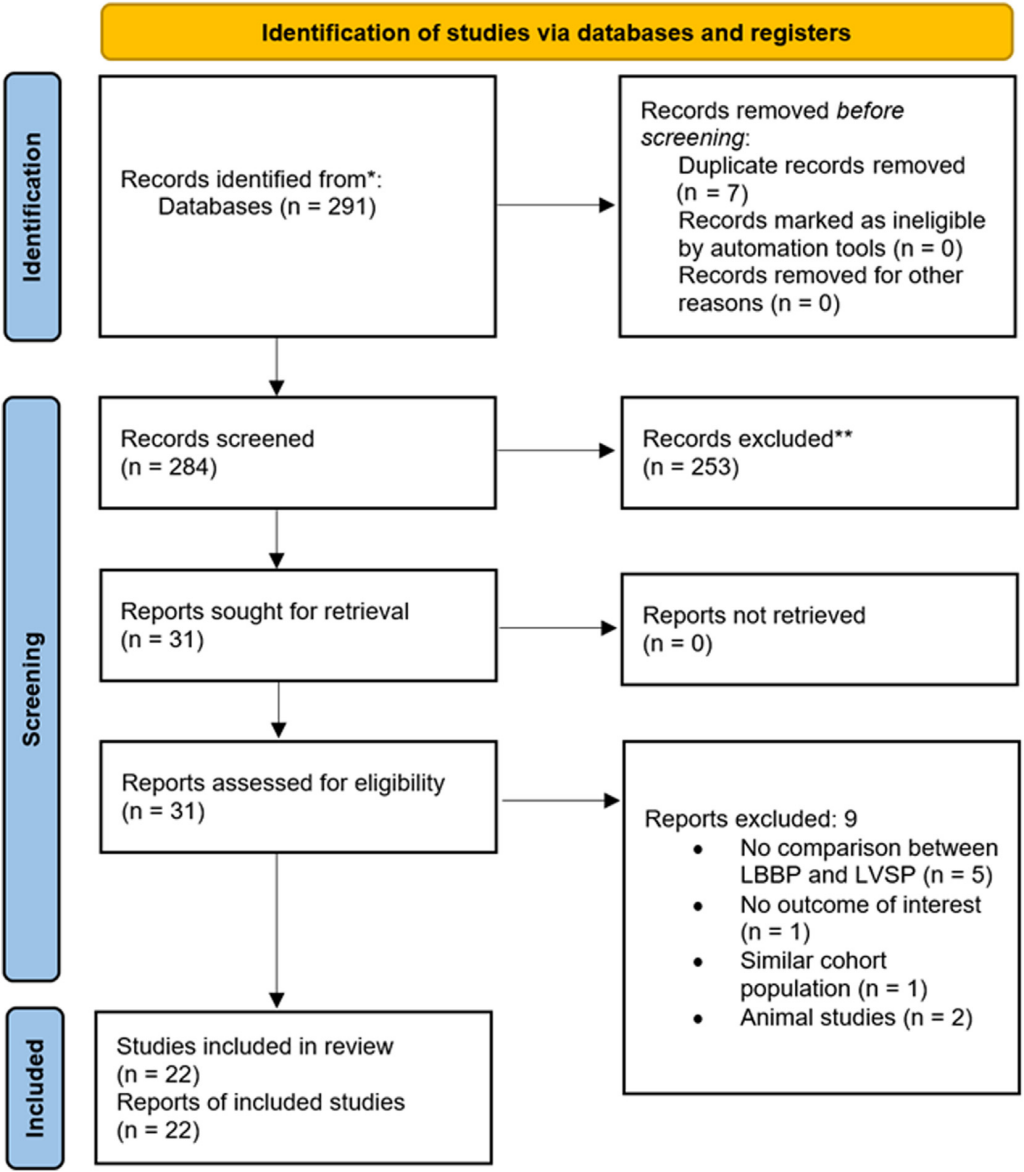
Flow diagram of study selection. LBBB = left bundle branch block; LVSP = left ventricular septal pacing.

The diagram of the selection process of included studies is presented in Figure 1. Initially, there were 291 studies collected from 3 databases. Seven duplicate studies have been excluded, followed by 253 studies excluded by title and abstract screening. Subsequently, 9 studies were excluded after full-text screening, resulting in 22 studies included in quantitative and qualitative analysis. All included studies had a NOS score of 7 or higher, indicating a low risk of bias, with a mean NOS of 7.6 ± 0.7 (Supplemental Table S1).

The baseline characteristics of the studies included in this analysis are presented in Table 1. A total of 22 studies were analyzed, comprising 12 retrospective cohort studies and 10 prospective cohort studies. These studies collectively involved 1360 LBBP and 1186 LVSP procedures. The primary indications for pacing across the studies were as follows: atrioventricular block (42.7%), CRT (33.4%), sinus node dysfunction (15.6%), AF with bradycardia (6.6%), bifascicular or trifascicular block (0.2%), atrioventricular node ablation (0.7%), PICM (0.1%), and other indications (0.7%). The mean age of participants was 73 ± 4 years, with 50.5% being male. The mean follow-up duration across studies was 15.1 ± 6.4 months. The pooled analyses for all variables are presented in Table 2.

**Table 1:** Baseline characteristics of included studies

Author (y), country	Study design	Total participants	Pacing indications (%)	Subgroup (%)	Pacing criteria	Age (mean ± SD)	Male n (%)	LVEF (mean ± SD)	QRS duration (mean ± SD)	Exclusion criteria	Mean follow-up period (mo)	Outcomes
Qian and colleagues^14^ (2021), China	Retrospective cohort	68	SND (32.3) AVB (36.8) Permanent AF with bradycardia (30.9)	LBBP (69.1) LVSP (30.9)	LBBP criteria: 1) Qr or QR pattern in lead V1 2) LBB potential recorded 3) LVAT <76 ms LVSP criteria: 1) Qr or QR pattern in lead V1 without LBB potential and LVAT ≥76 ms	67.7 ± 12.2	42 (61.8)	63.8 ± 3.0	116.9 ± 10.8	Patients with a history of cardiac device implantation	NS	Pacing parameters: QRS duration, LVAT
Zhang and colleagues^15^ (2021), China	Prospective cohort	103	SND (23.3) AVB (28.2) Persistent AF (41.7) Other (5.8)	Complete LBBP (20.4) Incomplete LBBP (55.3) LVSP (24.3)	Complete LBBP criteria: 1) RBBB pattern in V1 with LBB potential 2) Direct LBB capture: abrupt shortening of s-LVAT that was equal to the LBB po-LVAT and remained constant both at low and high outputs Incomplete LBBP criteria: 1) RBBB pattern in V1 with LBB potential 2) Incomplete LBB capture: longer s-LVAT at a low output and that could be shortened to LBB po-LVAT at high output (5 V at 0.5 ms) LVSP criteria: RBBB pattern in V1 with no LBB potential	71.2 ± 10	59 (57.3)	56 ± 15.5	108.9 ± 15.8	NS	6	Procedural parameters: Implant fluoroscopy time, procedural time, complications Pacing parameters: QRS duration, sensing amplitude, capture threshold, impedance, LVAT, ventricular pacing burden
Curila and colleagues^16^ (2021), The Netherlands	Prospective cohort	57	SND (29) AVB (59) Bifascicular/trifascicular block (8) AF with AV junctional ablation (3)	s-LBBP (40.3) ns-LBBP (100) LVSP (59.7)	s-LBBP criteria: 1) QRS pattern change to rsR or rSR pattern in lead V1 2) Unchanged V5 RWPT ns-LBBP criteria: Pseudo-RBB pattern with terminal r/R in lead V1 with pacing output 5 V at 0.5 ms, which changed to s-LBBP or LVSP while reducing the pacing outputs LVSP criteria: 1) QRS pattern change to terminal rs/Rs pattern in lead V1 2) Prolonged V5 RWPT >10 ms	77 ± 8	59 (61)	56 ± 6	NS	Patients with poor QRS signal quality did not allow for the construction of UHF-ECG maps	NS	Pacing parameters: s-LVAT
Wu and colleagues^17^ (2021), China	Prospective cohort	30	SND (13.3) AVB (26.7) CRT (36.7) Other (23.3)	s-LBBP (100) ns-LBBP (100) LVSP (100)	s-LBBP criteria: 1) QRS morphology changed to M or rsR’ pattern with wide R’ with a notch in lead V1 without the prolongation of s-LVAT 2) Only LBB capture identified ns-LBBP criteria: Qr or QR pattern in lead V1 with LBB and myocardial capture LVSP criteria: 1) QRS morphology changed with prolonged s-LVAT ≥10 ms by adjusting output within 1 V at 0.4 ms 2) No LBB capture identified	69.4 ± 9.5	16 (53)	49.3 ± 18.6	NS	Patients with infra-Hisian block and peripheral intraventricular conduction delay or ischemic cardiomyopathy with septal scar	NS	Pacing parameters: QRS duration, LVAT
Shimeno and colleagues^18^ (2021), Japan	Retrospective cohort	19	SND (42.1) AVB (57.9)	s-LBBP (100) ns-LBBP (100) LVSP (100)	s-LBBP criteria: 1) QRS morphology changed to M or rsR’ pattern with wide R’ with a notch in lead V1 without the prolongation of s-LVAT 2) Only LBB capture identified ns-LBBP criteria: Qr or QR pattern in lead V1 with LBB and myocardial capture LVSP criteria: 1) QRS morphology changed with prolonged s-LVAT ≥10 ms by adjusting output within 1 V at 0.4 ms 2) No LBB capture identified	79 ± 7	13 (68.4)	68 ± 6	109 ± 23	Patients who had insufficient recordings during the operation	NS	Pacing parameters: Capture threshold, s-LVAT
Heckman and colleagues^20^ (2021), The Netherlands	Prospective cohort	50	SND (28) AVB (36) Ablate and pace (10) Other (26)	s-LBBP (100) ns-LBBP (100) LVSP (100)	s-LBBP criteria: 1) Change in QRS morphology without a change in s-LVAT during decreasing the pacing output from ns-LBBP combined with an isoelectric interval between pacing spike and QRS complex 2) Only LBB capture identified ns-LBBP criteria: 1) Terminal r/R’ pattern in lead V1 2) Recording of an LBB potential during intrinsic rhythm (only in patients with normal ventricular activation) 3) Constant LVAT during high (8 V) and low (2 V) pacing output 4) The demonstration of transition from ns-LBBP to s-LBBP or ns-LBBP to LV myocardial only capture during decreasing pacing output LVSP criteria: 1) r’ present in lead V1 2) No LBB capture identified	74 ± 10	31 (61)	57 ± 7	113 ± 29	NS	NS	Pacing parameters: s-LVAT
Jastrzębski and colleagues^31^ (1) (2022), Poland	Prospective cohort	2533	SND (14.7) AVB (48.1) AF with bradycardia (3.7) CRT (27.5) Other (6)	LBBP (9) LVSP (21.5) LBFP (69.5)	LBBP criteria: Terminal R/r in lead V1 with at least 1 of the following LBB capture: 1) Pacing stimulus to V6RWPT <80 ms in patients with narrow QRS/patients with isolated RBBB or <90 ms in patients with more advanced ventricular conduction system disease 2) LBB potential to V6RWPT interval equal to the stimulus to V6RWPT interval (±10 ms) 3) V6–V1 interpeak interval >40 ms LVSP criteria: Terminal R/r in lead V1 with no LBB capture criteria	73.9 ± 11.8	1073 (42.4)	CRT candidate: 31.5% ± 8.3% Bradycardia: 57.3% ± 4.6%	141.5 ± 21.3	NS	6.4	Pacing parameters: QRS duration, sensing amplitude, capture threshold, V5/V6 RPWT
Jastrzębski and colleagues^19^ (2) (2022), Poland	Prospective cohort	124	SND (25.8) AVB (33.1) AF (10.5) CRT (30.6)	LBBP (57.3) ns-LBBP (100) LVSP (42.7)	s-LBBP criteria: 1) Transition from ns-LBB to s-LBB capture during a decrease in pacing output. ns-LBBP criteria: QR / rSR’ pattern in V1 with at least 1 of the following LBB capture: 1) Transition from ns-LBB to s-LBB capture during a decrease in pacing output 2) Transition from ns-LBB to LVS capture during programmed stimulation and/or burst/incremental pacing LVSP criteria: QR/rSR’ pattern in V1 without LBB capture	74.7 ± 11.4	71 (57.3)	46.2 ± 15.5	133.6 ± 34.8	Cases with LBB capture were confirmed only with some nondirect arbitrary criteria (LBB potential, V6RWPT, QRS duration, etc).	NS	Pacing parameters: QRS duration, V1–V6 interpeak interval
Zhou and colleagues^21^ (2022), China	Retrospective cohort	46	SND (34.8) AVB (58.7) PICM (6.5)	LBBP (50) LVSP (50)	LBBP criteria: RBBB pattern in lead V1 and at least 1 of the following LBB capture criteria 1. LVAT was abruptly shortened. 2. Paced QRS morphology was transitioned from nonselective to selective LBBP. LVSP criteria: RBBB pattern in V1 with no LBB capture	71.9 ± 9.3	23 (50)	58.89 ± 11.93	NS	NS	12	Procedural parameters: Procedural time Pacing parameters: QRS duration, sensing amplitude, capture threshold, impedance, LVAT Echocardiographic parameters: LVEF, LVEDD
Shimeno and colleagues^22^ (2022), Japan	Retrospective cohort	126	SND (30) AVB (70)	s-LBBP (51.9) ns-LBBP (100) LVSP (48.1)	s-LBBP criteria: 1) The QRS morphology change without the prolongation of the RWPT and the discrete local component separate from the stimulus artifact on the unipolar electrogram ns-LBBP criteria: 1) RBBB pattern in lead V1 2) V6 RWPT <90 ms and/or demonstration of LBB potential LVSP criteria: The QRS morphology change with prolongation of RWPT ≥10 ms owing to output drop within a 1.0 V difference	79 ± 11	59 (47)	63 ± 10	112 ± 29	NS	NS	Pacing parameters: QRS duration, capture threshold, V6 RPWT, V1–V6 interval peak
Qian and colleagues^24^ (2022), China	Retrospective cohort	105	SND (21.9) AVB (41.9) AF (16.2) CRT (20)	LBBP (76.3) LVSP (23.7)	LBBP criteria: QR or rSR pattern in V1 with at least 1 of the following conditions: 1) Transition from ns-LBB to s-LBB capture during decreasing pacing output 2) Abrupt shortening of LVAT >10 ms while changing pacing output 3) The difference between LVAT and the interval from intrinsic LBB potential to the R-wave peak in lead V5 or V6 was ≤5 ms if LBB potential could be recorded. Alternatively, the difference between the interval from the paced QRS onset to the R-wave peak and the interval from the intrinsic QRS onset to the R-wave peak in lead V5 or V6 was ≤5 ms if LBB potential could not be recorded. LVSP criteria: QR or QS pattern in V1 and none of the abovementioned 3 conditions were met.	70.3 ± 11	51 (50)	54.1 ± 4.9	HF: 162.6 ± 29.0	Incomplete ECG Unsuccessful recording of HB potential	NS	**Pacing parameters:** LVAT
Zhang and colleagues^32^ (2023), China	Prospective cohort	29	CRT (100)	LBBP (51.7) LVSP (48.3)	LBBP criteria: 1) RBBB pattern in lead V1 2) Abrupt shortening (≥10 ms) of s-LVAT in V6 with an increasing pacing output or constant and shortest Stim-LVAT at both high and low outputs 2) In patients with non-LBBB, the presence of LBB potential on electrogram was also needed to confirm LBB capture. LVSP criteria: RBBB pattern in V1 without other LBBP criteria	68.3 ± 9.8	14 (48.3)	32.2 ± 5.1	171.9 ± 14.5	NS	17	Pacing parameters: QRS duration, capture threshold, LVAT Echocardiographic parameters: LVEF, LVEDD Clinical outcome: HFH
Kato and colleagues^33^ (2023), Japan	Retrospective cohort	247	SND (25.9) AVB (70) AF and bradycardia (3.2) AV nodal ablation (0.8)	LBBP (54.7) LVSP (45.3)	LBBP criteria: Late R-wave in lead V1, s-LVAT <90 ms, and evidence of LBB capture: 1) Transition from nonselective LBBAP to selective LBBP with constant s-LVAT at high- and low-output pacing 2) Transition from nonselective LBBAP to LVSP by an abrupt increase in s-LVAT of >10 ms with decreasing pacing output 3) s-LVAT that was almost equal to the interval from LBB potential to peak R-wave in lead V6 during intrinsic conduction 4) Identification of retrograde HB electrogram during LBBAP using dual-lead technique LVSP criteria: Late R-wave in lead V1, s-LVAT <90 ms, and no evidence of LBB capture	79.6 ± 8.3	105 (42.5)	64.0 ± 9.8	117.0 ± 29.6	NS	24	Procedural parameters: Lead implantation time Pacing parameters: QRS duration, sensing amplitude, capture threshold, impedance, LVAT Echocardiographic parameters: LVEF
Peng and colleagues^23^ (2023), China	Retrospective cohort	59	SND (45.8) AVB (22) AF (15.3) AV node ablation (13.6) CRT (3.4)	LBBP (78) LVSP (22)	LBBP criteria: 1) RBBB pattern in V1 2) LBB potential was recorded during intrinsic rhythm or s-LVAT abruptly decreased ≥10 ms LVSP criteria: RBBB pattern in V1 with no LBB potential and Δ s-LVAT<10 ms	71.9 ± 12.0	21 (35.6)	63.9 ± 8.9	107.2 ± 24.0	Failed surgical procedures or development of severe complications, complete AVB with a ventricular escape rhythm, and complete LBBB	9	Pacing parameters: QRS duration, sensing amplitude, capture threshold, impedance, LVAT Clinical outcomes: HFH Echocardiographic parameters: LVEF, LVEDD
Shen and colleagues^26^ (1) (2023), China	Retrospective cohort	97	SND (34.1) AVB (59.8) AF (85) CRT (3.7)	s-LBBP (84.5) ns-LBBP (89.7) LVSP (9.3) RVSP (1)	s-LBBP criteria: 1) Paced QRS morphology changed with a transition from QR/qR to rsR/rSR/rSr in lead V1 2) Distinct isoelectric interval and discrete component appeared on EGM with a fixed Stim-V6RWPT ns-LBBP criteria: 1) Paced QRS morphology changed with a transition from Qr to QR/qR in lead V1 2) Stim-V6RWPT for 2 adjacent paced QRS complexes was abruptly decreased by ≥10 ms with a constant output (2 V/0.5 ms) during the process of lead screwing and remained shortest and constant at low and high outputs. LVSP criteria: Qr pattern in lead V1 without LBB capture criteria	73.9 ± 8.2	50 (61.0)	63.4 ± 10.5	NS	NS	NS	Pacing parameters: QRS duration, capture threshold, V6 RWPT
Shen and colleagues^34^ (2) (2025), China	Retrospective cohort	88	SND (31, 35.2) AVB (56, 63.6) AF bradycardia (4, 4.5) CRT (5, 5.7)	LBBP (100) ns-LBBP (100) LVSP (100)	s-LBBP criteria: 1) Paced QRS morphology changes with a transition in lead V1 and an isoelectric interval and discrete component appear on the EGM with fixed Stim-V6RWPT. ns-LBBP criteria: 1) Paced QRS morphology changes with a transition in lead V1 2) Stim-V6RWPT for 2 adjacent paced QRS complexes abruptly decreases by ≥10 ms with a constant output (2 V/0.5 ms) during the process of lead screwing and remains shortest and constant at low and high outputs. LVSP criteria: RBBB morphology in lead V1 without LBB capture	73.7 ± 9.1	51 (58)	62.8 ± 11.1	NS	Patients whose definitive LBB capture could not be confirmed	NS	Pacing parameters: Capture threshold
Cheng and colleagues^35^ (2024), China	Retrospective cohort	56	SND (5) AVB (16) AF and bradycardia (35)	LBBP (18) LVSP (22) LBFP (16)	LBBP criteria: RBBB pattern in lead V1 with evidence of LBB capture 1) ns-LBBP to s-LBBP with prolongation of V1 RWPT >15 ms 2) ns-LBBP to LVSP with prolongation of V6 RWPT >15 ms 3) Abrupt shortening of LVAT >10 ms or V6 RWPT <80 ms 4) Stim-V5 RWPT was equal to the LBB po-V5 RWPT if LBB potential was present. 5) Stim-V5 RWPT was equal to intrinsic V5 RWPT if LBB potential was not present. 6) V6–V1 interpeak interval >40 ms LVSP criteria: RBBB pattern in lead V1 with no evidence of LBB capture	75 ± 8.5	26 (46.4)	63.9 ± 9	107.6 ± 26.1	Patients with heart failure, LVEF <50%, severe valvular disease, history of valvular implantation or replacement, recent (<3 mo) myocardial infarction or myocardiopathy such as HCM and DCM	22.1	Pacing parameters: QRS duration, LVAT, V1 RPWT, V1–V6 interpeak interval Echocardiographic parameters: LVEF, LVEDD
Diaz and colleagues^25^ (2024), Colombia	Prospective cohort	415	CRT (100)	LBBP (34) LVSP (7.5) BiVP (58.5)	LBBP criteria: 1) Qr, qR, or rSR’ pattern in lead V1 2) s-LVAT was abruptly shortened by >10 ms. 3) Change in QRS duration and morphology when performing threshold testing or programmed stimulation 4) LVAT <80 ms 5) V6–V1 interpeak interval >44 ms LVSP criteria: Qr, qR, or rSR’ pattern in lead V1 without LBB capture	69.6 ± 11.3	290 (69.9)	26.2 ± 7.7	162 ± 25.3	Patients in whom conduction system pacing (either HB pacing or LBBAP) was undertaken as a bailout strategy to BiVP and those with a lack of response to previous CRT	13.4	Procedural parameters: Implant fluoroscopy time, procedural time, complications Pacing parameters: QRS duration, LVAT Echocardiographic parameters: LVEF Clinical outcomes: ACM or HFH, ACM, HFH
Cano and colleagues^27^ (2024), Spain	Retrospective cohort	323	SND (15, 4.7) AVB (226, 70) CRT (80, 25)	LBBP (161) LVSP (162)	LBBP criteria: RBBB pattern in lead V1 and at least 1 of the following LBB capture criteria 1. V6RWPT <80 ms 2. V1–V6 interpeak interval>33 ms 3. QRS transition (ns-LBBP to s-LBBP or ns-LBBP to LVSP) during pacing threshold or programmed ventricular stimulation 4. Sudden increase of V6RWPT >15 ms at reduced pacing output 5. Left bundle potential-V6RWPT = stim-V6RWPT (±10 ms) LVSP criteria: RBBB pattern in lead V1 without LBB capture	72 ± 15	197 (61)	52 ± 17	NS	First 100 patients considered as the learning curve	16	Procedural parameters: Lead implantation time, lead fluoroscopy time, complications Pacing parameters: QRS duration, sensing amplitude, capture threshold, impedance, ventricular pacing burden Echocardiographic parameters: LVEF
Rijks and colleagues^28^ (2024), The Netherlands	Prospective cohort	33	SND (4.11) AVB (7.19) Pace and ablate (19.51) CRT (7.19)	LBBP (21) LVSP (12)	LBBP criteria 1) Output-dependent transition in QRS morphology, either from nonselective to selective LBBP or from ns-LBBP to LVSP at decremental voltage output pacing 2) Interval from the left bundle potential to V6RWPT in intrinsic rhythm equal to pacing stimulus to V6RWPT (±10 ms) 3) Short and stable pacing stimulus to V6RWPT <75 ms in narrow baseline QRS and <80 ms in LBBB/interventricular conduction delay 4) V6–V1 interpeak time >44 ms **LVSP criteria:** 1) Paced qR/Qr complex in lead V1 without evidence of LBBP 2) Output-dependent transition from ns-LBBP to LVSP with decreasing output	73 ± 8	24 (65)	46 ± 11	117 ± 35	NS	NS	Pacing parameters: QRS duration, s-LVAT
Zhu and colleagues^29^ (2024), China	Prospective cohort	259	CRT (100)	LBBP (26.3) LVSP (14.7) BiVP (59)	LBBP criteria: qR, QR, rsR’, and rSR’ pattern in lead V1 with at least 1 of the following LBB capture criteria 1) LVAT was abruptly shortened by >10 ms. 2) Paced QRS morphology was transitioned from ns-LBBP to s-LBBP during decreasing outputs. 3) Paced QRS morphology was transitioned from ns-LBBP to LVSP (lengthening of LVAT by >10 ms when decreasing pacing outputs). LVSP criteria: Qr, qR, or QS pattern in lead V1 without LBB capture	63.1 ± 11.2	170 (65.6)	30.4 ± 6.9	165.9 ± 31.0	Age <18 y Patients with both LBBAP and LV optimized pacing (LOT-CRT) Pacemaker replacement with existing leads Patients receiving epicardial LV lead implantation for BiVP	28.8	Procedural parameters: Lead implantation time, procedural time, complications Pacing parameters: QRS duration, sensing amplitude, capture threshold, impedance, ventricular pacing burden, LVAT Echocardiographic parameters: LVEF, LVEDD Clinical outcomes: ACM or HFH, ACM, HFH
Chen and colleagues^30^ (2024), China	Retrospective cohort	91	CRT (100)	LBBP (57.1) LVSP (27.5) DSP (15.4)	LBBP criteria: Terminal r/R pattern in lead V1 and at least 1 of the following LBB capture criteria 1. s-LVAT <80 ms 2. LBB potential 3. QRS transition from ns-LBBP to s-LBBP LVSP criteria: RBBB pattern in V1 with no LBB capture	73.9 ± 10.8	61 (67)	35.9 ± 8.5	NS	NS	11.4	Procedural parameters: Implant fluoroscopy time, procedural time Pacing parameters: QRS duration, sensing amplitude, capture threshold, impedance, ventricular pacing burden, LVAT Echocardiographic parameters: LVEF, LVEDD Clinical outcomes: ACM or HFH, ACM, HFH

ACM = all-cause mortality; AF = atrial fibrillation; AV = atrioventricular; AVB = atrioventricular block; BiVP = biventricular pacing; CRT = cardiac resynchronization therapy; DCM = dilated cardiomyopathy; DSP = deep septal pacing; ECG = electrocardiogram; EGM = intracardiac electrogram; HB = His bundle; HCM = hypertrophic cardiomyopathy; HF = heart failure; HFH = heart failure hospitalization; LBB = left bundle branch; LBBAP = left bundle branch area pacing; LBBB = left bundle branch block; LBB po-LVAT = left bundle branch potential to left ventricular activation time; LBBP = left bundle branch pacing; LBFP = left bundle fascicular pacing; LVAT = left ventricular activation time; LVEDD = left ventricular end-diastolic diameter; LVEF = left ventricular ejection fraction; LVS = left ventricular septal; LVSP = left ventricular septal pacing; NS = not specified; ns-LBBP = nonspecific left bundle branch block; PICM = pacing-induced cardiomyopathy; RBBB = right bundle branch block; RVSP = right ventricular septal pacing; RWPT = R-wave peak time; SD = standard deviation; s-LVAT = stimulus-to-left ventricular activation time; s-LBBP = specific left bundle branch pacing; SND = sinus node dysfunction; UHF-ECG = ultrahigh-frequency electrocardiogram.

**Table 2:** Summary of the pooled effect sizes for all outcome variables

Numerical variables				
Variables	MD	95% CI	I^2^	*P* value
Procedural outcomes				
- Procedural time	2.76 min	−18.94 to 24.47	88.2%	.803
- Implant fluoroscopy time	0.27 min	−4.33 to 4.86	83.8%	.91
- Lead implantation time	−6.03 min	−15.72 to 3.65	90.3%	.222
Pacing parameters postoperatively				
- Pacing sensing amplitude	0.03 mV	−0.64 to 0.69	13.7%	.936
- Pacing capture threshold	−0.01 V	−0.06 to 0.04	41.4%	.63
- Pacing impedance	−23.52 Ω	−72.35 to 25.3	71.2%	.345
Pacing parameters during follow-up				
- Pacing sensing amplitude	0.80 mV	−0.45 to 2.05	43.7%	.209
- Pacing capture threshold	0.02 V	−0.09 to 0.12	54.2%	.776
- Pacing impedance	−5.38 Ω	−56.58 to 45.82	85.7%	.837
Paced QRS duration	−9.65 ms	−13.35 to −5.96	84.9%	<.001∗
Subgroup analysis				
-Narrow QRS duration	−7.49 ms	−10.44 to −4.53	36.7%	<.001∗
-Wide QRS duration	−16.27 ms	−23.45 to −9.09	80.5%	<.001∗
Sensitivity analysis				
-Wide QRS duration	−12.07 ms	−15.96 to −8.19	18.4%	<.001∗
Reduction in paced QRS duration				
Subgroup analysis				
Wide QRS duration				
-LBBP	22.57 ms	1.49–43.65	97.4%	.036∗
-LVSP	5.78 ms	−11.48 to 23.05	94%	.511
Baseline QRS duration >150 ms				
-LBBP	38.87 ms	31.57–46.17	62%	<.001∗
-LVSP	19.71 ms	13.23–26.2	0%	<.001∗
-LBBP vs LVSP	20.82 ms	13.07–28.56	0%	<.001∗
Narrow QRS duration				
-LBBP	−6.28 ms	−13.05 to 0.48	71.9%	.069
-LVSP	−13.77 ms	−20.43 to −7.11	56.3%	.001∗
s-LVAT	−14.62 ms	−16.99 to −12.24	77.2%	<.001∗
Subgroup analysis				
-Narrow QRS duration	−10.24 ms	−12.21 to −8.28	25.6%	<.001∗
-Wide QRS duration	−18.04 ms	−21.90 to −14.19	60.8%	<.001∗
Sensitivity analysis				
-Wide QRS duration	−17.6 ms	−22.7 to −12.51	44.4%	<.001∗
V1 RWPT	3.91 ms	−3.12 to 10.94	79.40%	.275
V1−V6 interpeak interval	16.25 ms	2.95–29.54	98.9%	.017∗
Sensitivity analysis	5.87 ms	3.63–8.39	0%	<.001∗
Ventricular pacing burden	0.32 %	−4.79 to 5.43	85.70%	.901
Subgroup based on pacing location				
s-LBBP vs LVSP				
-s-LVAT	−15.1 ms	−16.6 to −13.6	0%	<.001∗
-Paced QRS duration	−10.55 ms	−15.26 to −5.84	0%	<.001∗
-V1 RWPT	9.32 ms	3.2–15.4	62.9%	.003∗
-V1−V6 interpeak interval	31.63 ms	23.02–40.24	73.4%	<.001∗
ns-LBBP vs LVSP				
-s-LVAT	−14.88 ms	−18.36 to −11.4	78.8%	<.001∗
-Paced QRS duration	−6.31 ms	−10.92 to −1.69	0%	.007∗
-V1 RWPT	−3.51 ms	−6.39 to 0.64	0%	.017∗
-V1−V6 interpeak interval	13.82 ms	10.99–16.64	0%	<.001∗
s-LBBP vs ns-LBBP				
-s-LVAT	−0.16 ms	−2.15 to 1.83	58.6%	.875
-Paced QRS duration	−4.31 ms	−8.35 to −0.27	0%	.037∗
-V1 RWPT	13.32 ms	4.99–21.63	81.3%	.002∗
-V1−V6 interpeak interval	18.2 ms	12.3–24.04	48.7%	<.001∗
LVEF difference in the LBBP group				
-Reduced LVEF	14%	8.71–19.29	93.4%	<.001∗
-Preserved LVEF	−0.91%	−2.58 to 0.76	0%	.284
Sensitivity analysis				
-Reduced LVEF	11.33%	9.99–12.67	0%	<.001∗
LVEF difference in the LVSP group				
-Reduced LVEF	6.48%	3.64–9.32	44.7%	<.001∗
-Preserved LVEF	−0.51%	−2.48 to 1.46	0%	.612
Sensitivity analysis				
-Reduced LVEF	5.54%	3.17–7.91	22.8%	<.001∗
Increase in LVEF between LBBP and LVSP				
-Reduced LVEF	6.05%	2.9–9.2	51.3%	<.001∗
-Preserved LVEF	0.36%	−2.30 to 3.02	0%	.789
Sensitivity analysis				
-Reduced LVEF	5.26%	2.59–7.93	33.1%	<.001∗
LVEDD difference in the LBBP group				
-Large LVEDD	−7.28 mm	−11.04 to −3.51	75%	<.001∗
-Small LVEDD	−0.21 mm	−1.75 to 1.34	0%	.794
Sensitivity analysis				
-Large LVEDD	−5.46 mm	−7.26 to −3.65	0%	<.001∗
LVEDD difference in the LVSP group				
-Large LVEDD	−3.56 mm	−6.3 to −0.81	0%	.011∗
-Small LVEDD	−0.22 mm	−1.85 to 1.42	0%	.796
Reduction in LVEDD between LBBP and LVSP				
-Large LVEDD	2.63 mm	−0.63 to 5.9	0%	.114
-Small LVEDD	−0.03 mm	−2.28 to 2.22	0%	.978

Categorical variables				

Variables	RR	95% CI	I^2^	*P* value

Procedural complications				
- Ventricular septal perforation	1.79	0.55–5.83	20.2%	.336
- Lead dislodgement	0.14	0.02–1.08	0%	.06
All-cause mortality and/or HFH	0.39	0.26–0.57	55.1%	<.001∗
All-cause mortality	0.38	0.18–0.77	57.1%	.007∗
HFH				
Subgroup analysis	0.32	0.19–0.57	18.1%	<.001∗
- Reduced LVEF and wide QRS duration	0.30	0.14–0.64	49.9%	.002∗
- Preserved LVEF and narrow QRS duration	0.50	0.09–2.87	0%	.440
All-cause mortality and/or HFH (adjusted)	0.28	0.17–0.48	0%	<.001∗
All-cause mortality (adjusted)	0.38	0.17–0.88	73.5%	.023∗

CI = confidence interval; HFH = heart failure hospitalization; I^2^ = heterogeneity index; LBBP = left bundle branch pacing; LVEDD = left ventricular end-diastolic diameter; LVEF = left ventricular ejection fraction; LVSP = left ventricular septal pacing; MD = mean difference; ns-LBBP = nonspecific left bundle branch pacing; RR = risk ratio; RWPT = R-wave peak time; s-LBBP = specific left bundle branch pacing; s-LVAT = stimulus-to-left ventricular activation time.

∗ Significant *P* value.

### Meta-analysis of procedural outcomes

Seven studies were included in the analysis to evaluate procedural outcomes.^15^^,^^21^^,^^25^^,^^27^^,^^29^^,^^30^^,^^33^ No significant differences were observed between the 2 groups for procedural time, implant fluoroscopy time, and lead implantation time (Supplemental Figure S1). Regarding complications, the risk of ventricular septal perforation and lead dislodgement did not differ significantly between the 2 groups (Supplemental Figure S2).

### Meta-analysis of electrophysiological outcomes

Twenty studies were included in the analysis to assess electrophysiological outcomes.^14^, ^15^, ^16^, ^17^, ^18^, ^19^, ^20^, ^21^, ^22^, ^23^, ^24^, ^25^, ^26^, ^27^, ^28^, ^29^, ^30^^,^^33^, ^34^, ^35^ No significant differences were observed between the LBBP and LVSP groups regarding pacing sensing amplitude, pacing capture threshold, or pacing impedance, both postoperatively and during follow-up (Supplemental Figures S3 and S4).

**Figure 2:**
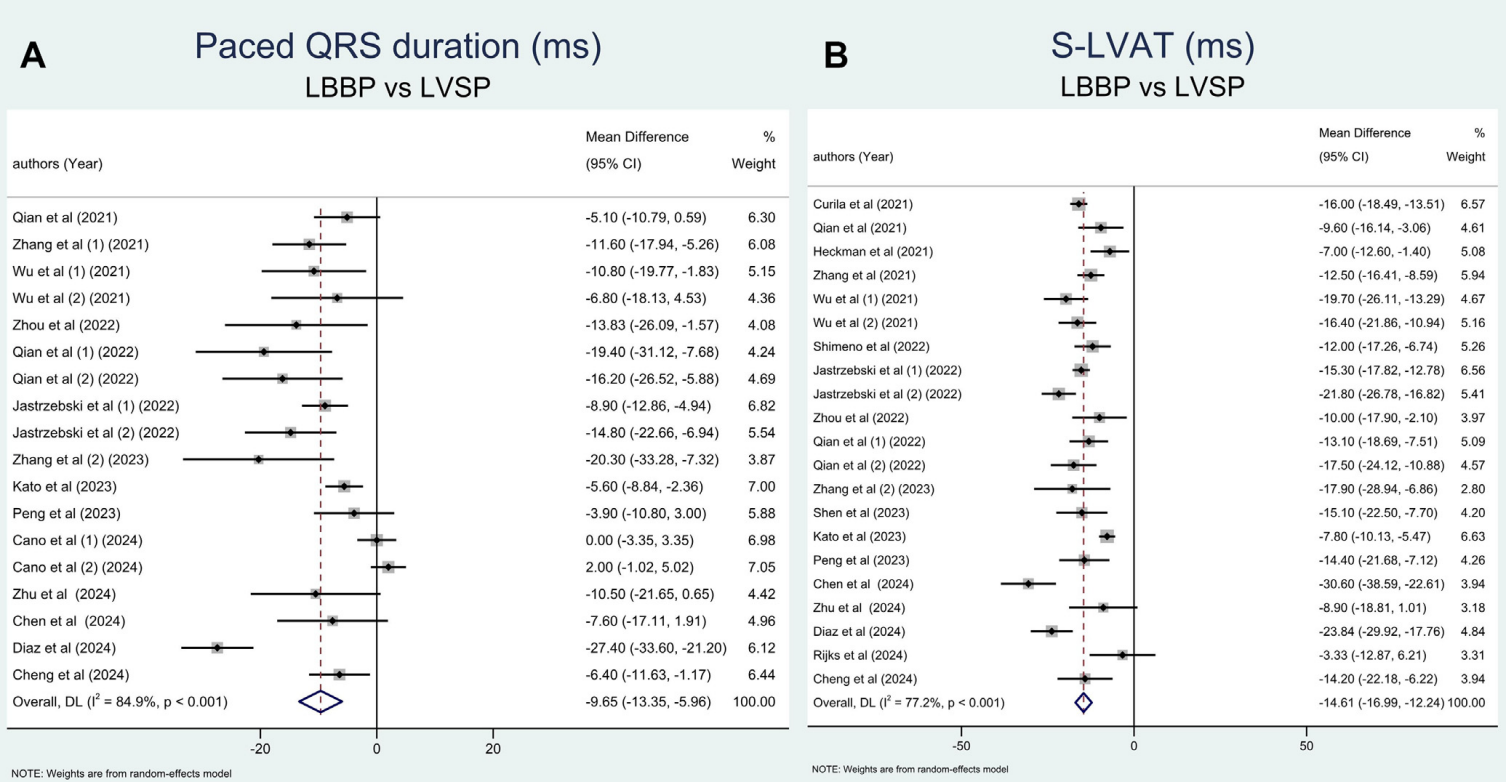
Comparison of paced QRS duration and s-LVAT between LBBP and LVSP. **A:** Paced QRS duration (milliseconds). **B:** s-LVAT (milliseconds). CI = confidence interval; LBBP = left bundle branch pacing; LVSP = left ventricular septal pacing; s-LVAT = stimulus-to-left ventricular activation time.

The paced QRS duration was significantly shorter in the LBBP group (Figure 2). Given the high heterogeneity, a subgroup analysis was conducted based on baseline QRS duration, which significantly reduced the heterogeneity. Three studies were excluded from this analysis owing to the absence of baseline paced QRS duration data.^17^^,^^27^^,^^30^ The subgroup analysis revealed that the paced QRS duration remained significantly shorter in the LBBP group across 2 subgroups: narrow QRS duration and wide QRS duration (Supplemental Figure S5). Given the high heterogeneity in the subgroup with baseline QRS of >120 ms, a sensitivity analysis was performed by excluding the study by Diaz et al,^25^ which significantly reduced the heterogeneity to 0%, while maintaining statistical significance (Supplemental Figure S5). Notably, the study by Diaz et al^25^ incorporated additional LBB capture criteria, including LVAT of <80 ms and a V6–V1 interpeak interval of >44 ms, which may explain the discrepancies in pacing subgroup categorization and its impact on paced QRS duration outcomes.

To assess the impact of pacemaker implantation on native QRS duration, a subgroup analysis was conducted based on baseline QRS durations. In studies with wide QRS duration, a significant reduction in paced QRS duration after pacemaker implantation was observed exclusively in the LBBP group (Supplemental Figure S6). Further analysis of studies with baseline QRS durations of >150 ms revealed significant reductions in native QRS duration in both groups. Moreover, the LBBP group exhibited a significantly greater reduction in native QRS duration than the LVSP group (Supplemental Figure S7).

In studies with baseline narrow QRS duration, the analysis showed no significant change in QRS duration in the LBBP group after pacemaker implantation. However, in the LVSP group, there was a significant increase in QRS duration after pacemaker implantation (Supplemental Figure S8).

s-LVAT was significantly shorter in the LBBP group (Figure 2). Owing to high heterogeneity, a subgroup analysis was performed based on baseline QRS duration and significantly reduced the heterogeneity. Four included studies were excluded from the subgroup analysis because baseline QRS duration was not mentioned in these studies.^16^^,^^17^^,^^26^^,^^30^ The subgroup analysis showed that s-LVAT was substantially shorter in the LBBP group in subgroups of narrow and wide QRS duration (Supplemental Figure S9). However, given that high heterogeneity was still noted in the subgroup analysis of wide QRS duration, sensitivity analysis was performed by excluding Diaz et al’s^25^ and Jastrzębski et al’s^31^ studies, which resulted in reduced heterogeneity to 44.4% while maintaining its significance (Supplemental Figure S9). Among all studies in the subgroup of wide QRS duration, Diaz et al^25^ and Jastrzębski et al^31^ used additional LBB capture criteria diagnosis including LVAT of <80–90 ms and a V6–V1 interpeak interval of >40–44 ms, leading to differences in QRS duration outcome.

V1 RWPT was not significantly different in both groups (Supplemental Figure S10). V1–V6 interpeak interval was significantly longer in the LBBP group (Supplemental Figure S10). Owing to high heterogeneity, sensitivity analysis was performed by excluding Shimeno et al’s^22^ and Jastrzębski et al’s^31^ studies because these 2 studies only used 1 pacing criterion including transition from nonspecific LBBP (ns-LBBP) to specific LBBP (s-LBB) or LVS capture during changing the pacing output. Sensitivity analysis resulted in a substantial reduction of heterogeneity to 0% while maintaining its significance (Supplemental Figure S10). Finally, ventricular pacing burden did not differ significantly between the 2 groups (Supplemental Figure S11).

### Subgroup analysis based on pacing location

There are 7 studies that further classified LBBP into s-LBBP and ns-LBBP.^15^, ^16^, ^17^^,^^19^^,^^22^^,^^26^^,^^34^

### s-LBBP vs LVSP

s-LVAT and paced QRS duration were significantly shorter in the s-LBBP group. In contrast, V1 RWPT and V1–V6 interpeak interval were significantly longer in the s-LBBP group (Supplemental Figure S12).

### ns-LBBP vs LVSP

s-LVAT, paced QRS duration, and V1 RWPT were significantly shorter in the ns-LBBP group. Contrastingly, V1–V6 interpeak interval significantly longer in the ns-LBBP group (Supplemental Figure S13).

### s-LBBP vs ns-LBBP

s-LVAT was not significantly different in both groups. Paced QRS duration was significantly shorter in the s-LBBP group. V1 RWPT and V1–V6 interpeak interval were significantly longer in the s-LBBP group (Supplemental Figure S14).

### Meta-analysis of echocardiographic outcomes

#### LVEF

**Figure 3:**
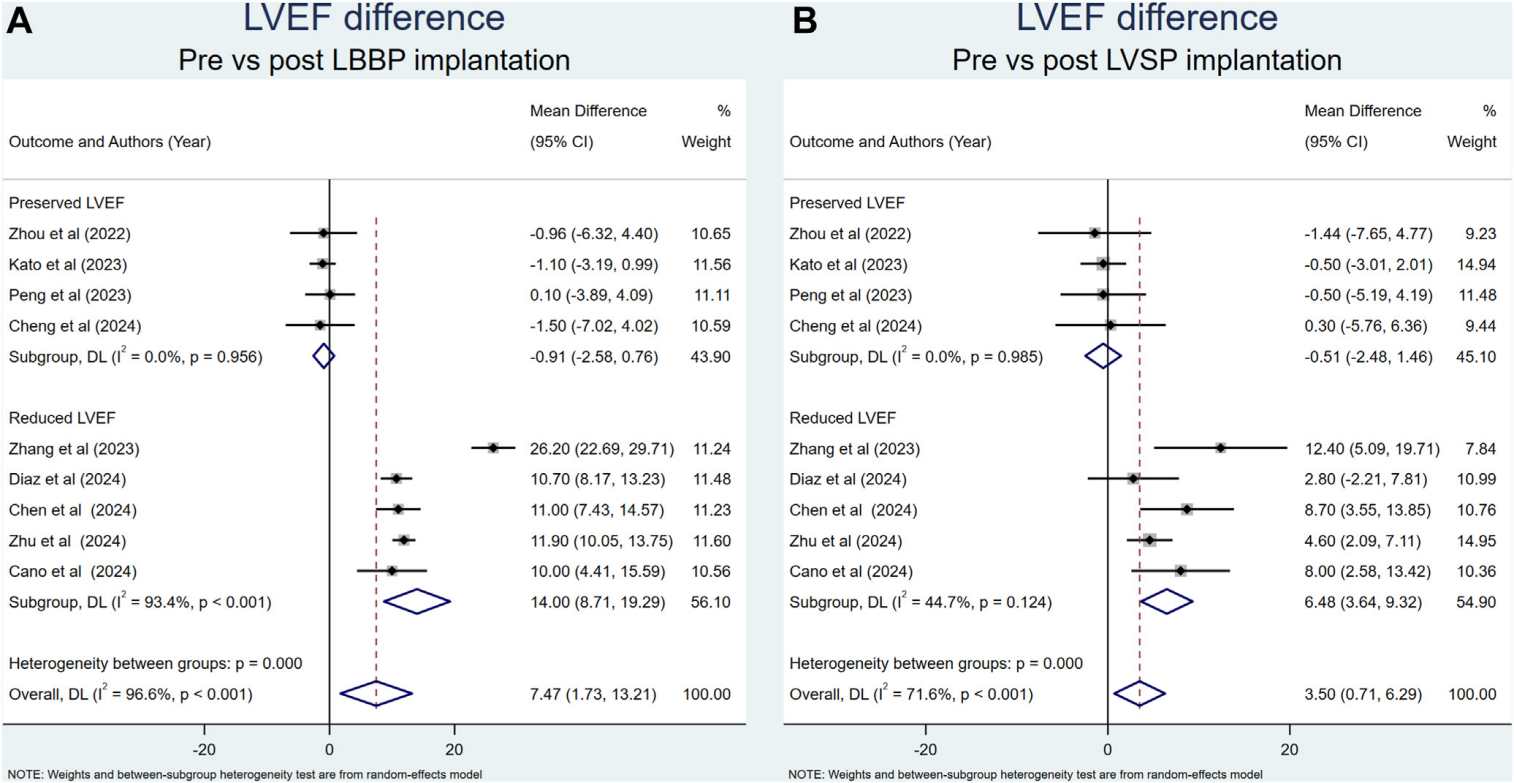
Comparison of LVEF before and after pacemaker implantation. **A:** LBBP. **B:** LVSP. CI = confidence interval; LBBP = left bundle branch pacing; LVEF = left ventricular ejection fraction; LVSP = left ventricular septal pacing.

A total of 9 studies were included in the analysis of echocardiographic outcomes.^21^^,^^23^^,^^25^^,^^27^^,^^29^^,^^30^^,^^32^^,^^33^^,^^35^ After LBBP implantation, LVEF was only significantly increased in patients with reduced LVEF (Figure 3). Sensitivity analysis was performed by excluding Zhang et al’s^15^ study because, in contrast to other included studies, Zhang et al^15^ used only 1 LBB capture criterion. Sensitivity analysis showed a significant reduction of the heterogeneity index to 0% while maintaining its significance (Supplemental Figure S15). Similarly, LVEF was only significantly increased after LVSP implantation in patients with reduced LVEF (Figure 3). Sensitivity analysis by excluding Zhang et al’s^15^ study resulted in a substantial reduction of the heterogeneity index to 22.8% while maintaining its significance (Supplemental Figure S15). The increase in LVEF was significantly greater in the LBBP group than the LVSP group for patients with reduced LVEF (Supplemental Figure S16). Sensitivity analysis by excluding Zhang et al’s study^15^ resulted in a substantial reduction of the heterogeneity index to 33.1% while maintaining its significance (Supplemental Figure S15).

#### LVEDD

**Figure 4:**
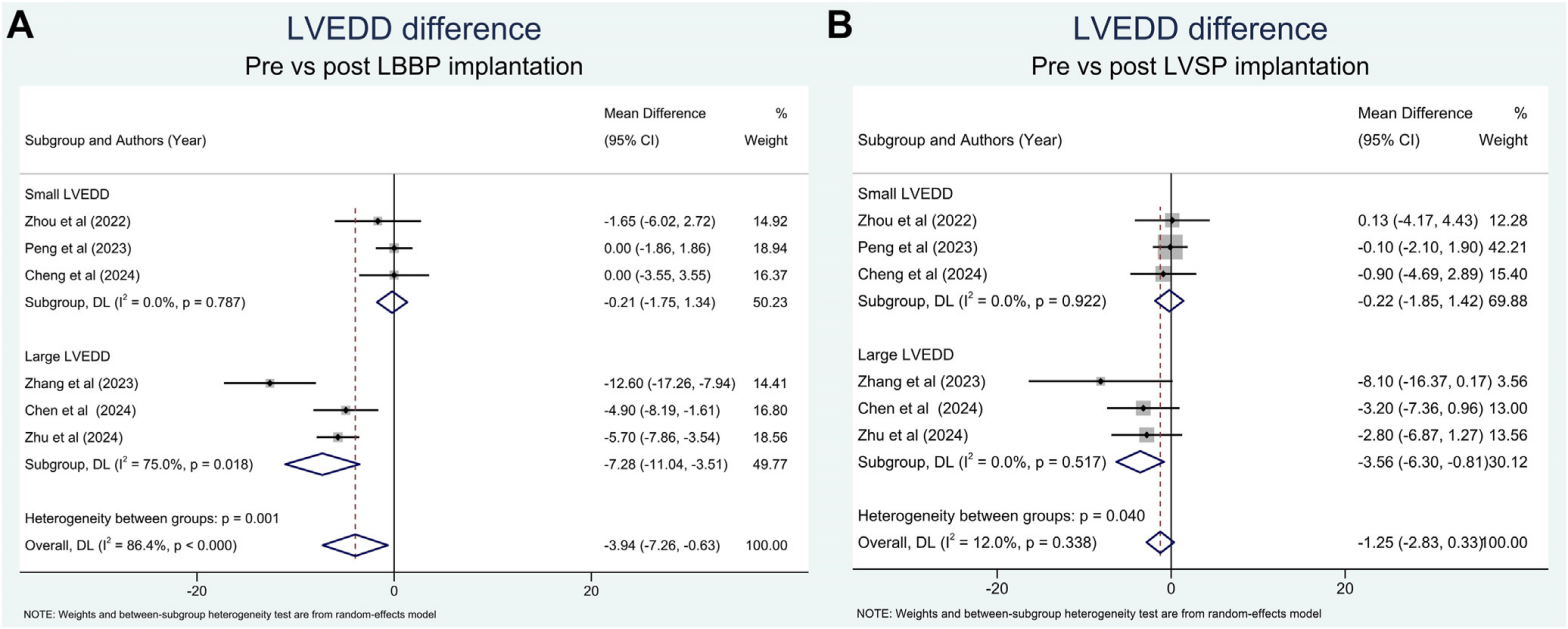
Comparison of LVEDD before and after pacemaker implantation. **B:** LBBP. **B:** LVSP. CI = confidence interval; LBBP = left bundle branch pacing; LVEDD = left ventricular end-diastolic diameter; LVSP = left ventricular septal pacing.

A significant reduction of LVEDD after LBBP implantation was only observed in patients with large LVEDD (Figure 4). Sensitivity analysis by excluding Zhang et al’s^15^ study resulted in a substantial reduction of the heterogeneity index to 0% while maintaining its significance (Supplemental Figure S17). LVEDD was only significantly reduced after LVSP implantation in patients with large LVEDD (Figure 4). There was no significant difference in the reduction of LVEDD between the 2 groups for patients with large LVEDD and small LVEDD (Supplemental Figure S18).

### Meta-analysis of clinical outcomes

**Figure 5:**
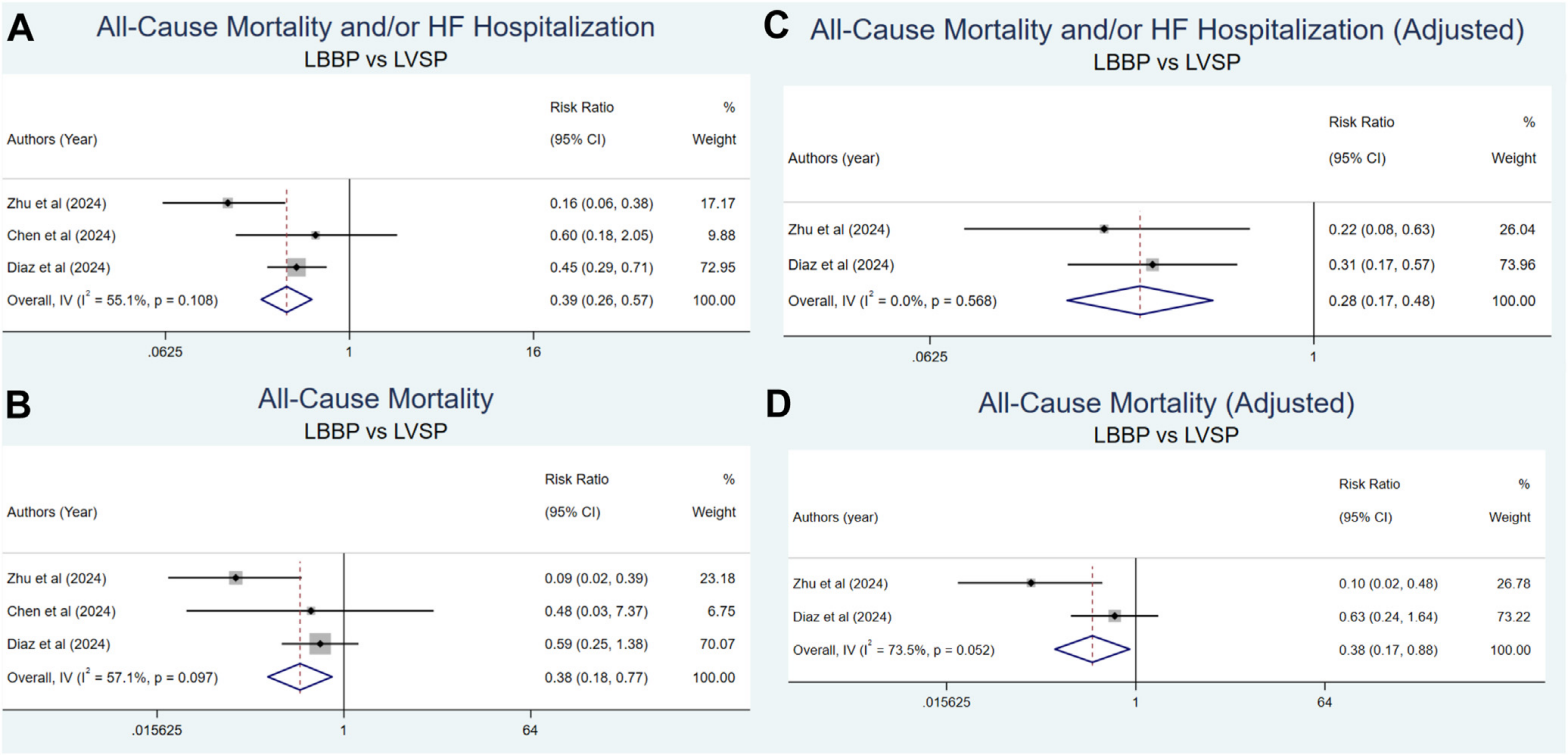
Comparison of all-cause mortality and HFH between LBBP and LVSP. **A:** All-cause mortality and/or HFH (unadjusted). **B:** All-cause mortality (unadjusted). **C:** All-cause mortality and/or HFH (adjusted). **D:** All-cause mortality (adjusted). CI = confidence interval; HFH = heart failure hospitalization; LBBP = left bundle branch pacing; LVSP = left ventricular septal pacing.

**Figure 6:**
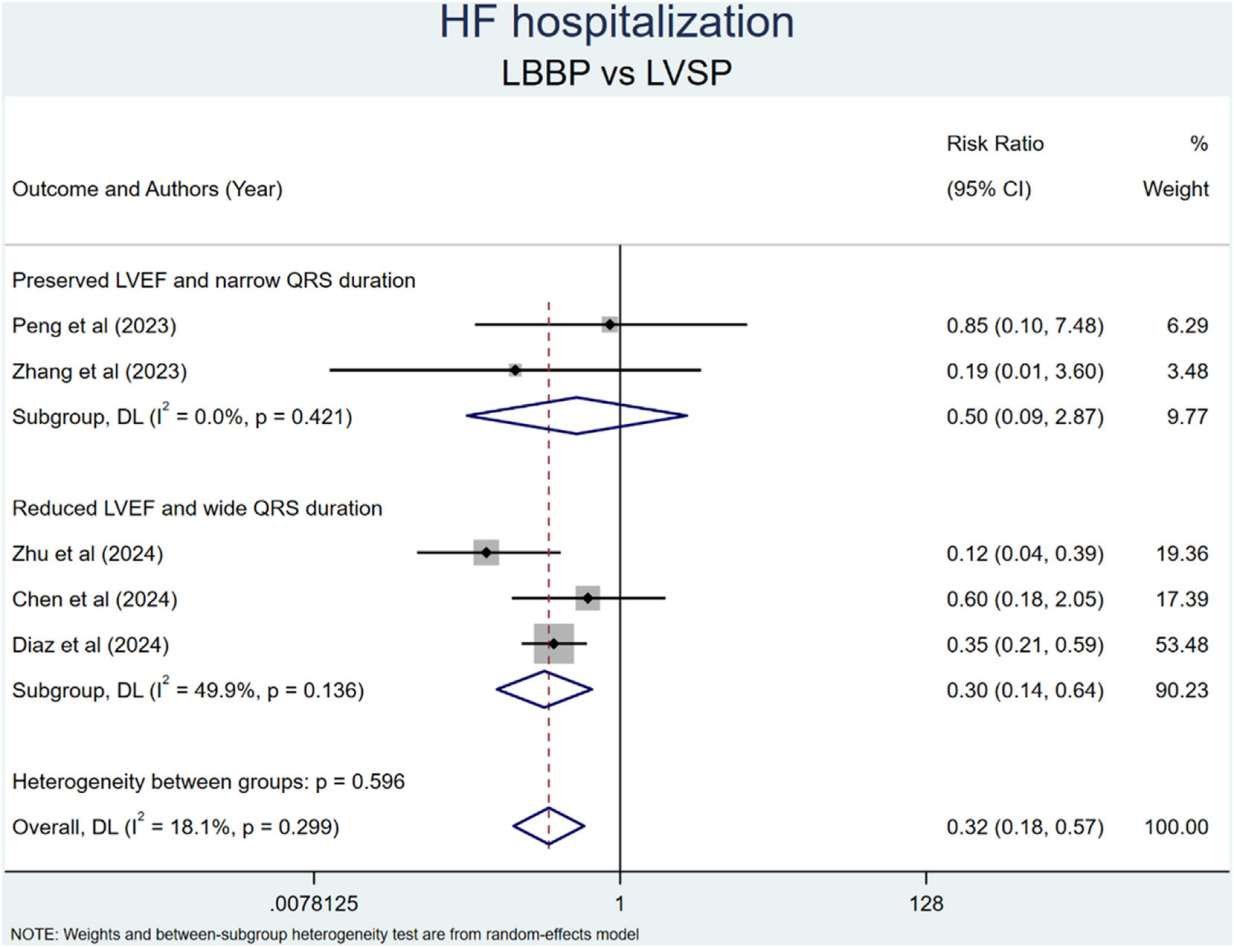
Comparison of HFH between LBBP and LVSP. CI = confidence interval; HFH = heart failure hospitalization; LBBP = left bundle branch pacing; LVEF = left ventricular ejection fraction; LVSP = left ventricular septal pacing.

Regarding clinical outcomes, 5 studies were included in the analysis.^23^^,^^25^^,^^29^^,^^30^^,^^32^ This meta-analysis demonstrated that the LBBP group significantly reduced the risk of all-cause mortality and/or HFH compared with the LVSP group. In addition, when analyzed separately, the LBBP group showed a significant reduction in the risk of all-cause mortality and HFH compared with the LVSP group (Figures 5 and 6).

A subgroup analysis was performed to evaluate HFH outcomes in patients with preserved LVEF and narrow QRS duration and those with reduced LVEF and wide QRS duration. The analysis showed that the LBBP group had a significantly lower risk of HFH than the LVSP group, specifically in patients with reduced LVEF and wide QRS duration (Figure 6). However, subgroup analyses were not performed for all-cause mortality or HFH outcomes, because all included studies exclusively comprised participants with reduced LVEF and wide QRS duration.

After adjusting for several confounding factors across the included studies, the meta-analysis revealed that the LBBP group independently and significantly reduced the risk of all-cause mortality and/or HFH and all-cause mortality compared with the LVSP group (Figure 5).

### Publication bias

Begg’s funnel plot analysis demonstrated visually symmetrical plots, and no evidence of small-study effects was observed in Egger’s test for the comparison of LBBP and LVSP with regard to paced QRS duration and LVAT (*P* > .05).

## Discussion

In summary, this meta-analysis yielded several key findings. First, intraventricular synchrony was better preserved in the LBBP group, as evidenced by a shorter s-LVAT. Second, interventricular synchrony was more effectively maintained in the LVSP group, as indicated by a shorter V1–V6 interpeak interval. Third, in patients with reduced LVEF and wide QRS duration, the reduction in native QRS duration was significantly greater in the LBBP group, which was associated with greater improvements in LVEF and LVEDD and a reduction in the risk of all-cause mortality and HFH. Fourth, in patients with preserved LVEF and narrow QRS duration, the prolongation of native QRS duration was more pronounced in the LVSP group, although there were no significant differences in LVEF, LVEDD, or the risk of HFH between the 2 groups. Finally, there were no significant differences in procedural duration or complications between the 2 groups. In addition, pacing parameters, including sensing amplitude, capture threshold, pacing impedance, and V1 RWPT, were comparable between the groups.

### Pacing properties, procedural complications, and duration

This meta-analysis showed that the pacing capture threshold, pacing impedance, and ventricular pacing burden percentage were not significantly different between the 2 groups, denoting that the battery lifetime was similar between the 2 groups.

In addition, the success of LBBP implantation is influenced by various factors, including a thickened or fibrous septum and the failure to meet the LBB capture criteria, which can result from electrical abnormalities in the ventricle, such as IVCD.^33^^,^^45^, ^46^, ^47^ Hence, owing to its deeper implantation site and the variety of factors influencing the success of LBB capture, LBBP implantation is generally considered more complex and time consuming than LVSP implantation. However, this meta-analysis found no significant differences in procedural time, lead implantation time, or fluoroscopy time between the 2 groups, indicating that LBBP is safe and not excessively time consuming. In addition, this meta-analysis found no significant difference in the rates of septal perforation or lead dislodgement between LBBP and LVSP.

### Intraventricular synchrony

Theoretically, because LBBP directly stimulates the LBB, it is expected to result in a shorter LVAT, given that it promotes physiological LV excitation. In contrast, LVSP indirectly activates the LBB by first stimulating the left septal myocardial cells, leading to a longer LVAT.^48^ Consistent with this, Chen et al^49^ reported that, in the LBBP group, the LV electrical activation sequence, as detected via coronary sinus electrograms, mirrored the intrinsic rhythm, with depolarization spreading from the lateral to the posterior wall. In contrast, the LVSP group exhibited a nonphysiological depolarization pattern, with activation progressing from the posterior to the lateral wall. This meta-analysis confirmed that the LBBP group significantly reduced s-LVAT by 14.5 ms compared with the LVSP group, with this difference remaining statistically significant in both patients with narrow and wide baseline QRS complexes.

The preservation of LV electrical synchrony by LBBP has also been shown to result in the preservation of LV mechanical synchrony, as confirmed by several studies. Qian et al^14^ demonstrated that shorter LVAT was associated with improved LV mechanical synchrony, as assessed by electrocardiogram-gated single-photon emission computed tomography imaging. Furthermore, Zhang et al^32^ found that tissue synchronization imaging of 12 LV segments revealed significantly shorter activation times in the LBBP group than the LVSP group, indicating better intraventricular mechanical synchrony in LBBP. Collectively, these findings reinforce the conclusion that LBBP results in superior LV electrical and mechanical synchrony compared with LVSP.

It is hypothesized that s-LBBP leads to a shorter s-LVAT than ns-LBBP, because s-LBBP targets only the LBB, whereas ns-LBBP captures both the myocardial septum and LBB.^15^, ^16^, ^17^^,^^19^^,^^22^^,^^26^^,^^34^ However, this meta-analysis found no significant difference in s-LVAT between the s-LBBP and ns-LBBP groups. Furthermore, s-LVAT in both s-LBBP and ns-LBBP groups were significantly shorter than the LVSP group. Therefore, it is unlikely that variations in the location of the LBBP lead tip will affect the s-LVAT outcomes in this study.

In contrast, s-LVAT might be influenced by the proximity of the LVSP lead tip to the LBB. Theoretically, a shorter distance between the lead tip and the LBB would result in reduced depolarization time between the activated left septal myocardial cells and the LBB, thereby shortening the LVAT.^16^ Supporting this concept, Curila et al^16^^,^^50^ demonstrated that positioning the LVSP lead closer to the LBB resulted in a decrease in LVAT from 86.3 ± 3.8 ms to 68 ± 4.6 ms. This change was not significantly different from the LVAT observed in the LBBP group. Unfortunately, given the current diagnostic criteria for LVSP, it is not possible to precisely determine the location of the LVSP lead tip in relation to the LBB. Thus, variations in ventricular depolarization time may still occur.

### Interventricular synchrony

LBBP has been proposed to induce interventricular dyssynchrony, whereas LVSP has been shown to preserve interventricular synchrony.^51^ LBBP accelerates LV activation but delays RV activation owing to the longer retrograde conduction time from LBB to the right bundle branch, whereas LVSP enables simultaneous LV and RV activation owing to its direct left-to-right septal activation.^19^^,^^34^^,^^51^ However, this meta-analysis found no significant difference in V1 RWPT between the 2 groups, indicating similar RV depolarization duration. However, variations in LBBP diagnostic criteria and preexisting bundle branch block or IVCD may explain these findings.^19^^,^^34^ The subgroup analysis showed that s-LBBP had a longer V1 RWPT than both LVSP and ns-LBBP groups, indicating that diagnostic criteria variability affects these results. Furthermore, the V1–V6 interpeak interval was significantly longer in the LBBP group, confirming better interventricular synchrony preservation in the LVSP group.

However, Zhang et al^32^ demonstrated that interventricular mechanical delay, assessed using pulsed-wave Doppler, did not differ significantly between the LBBP and LVSP groups. Therefore, we hypothesize that the modest prolongation of the V6–V1 interpeak interval (approximately 6 ms) observed in this meta-analysis may not be sufficiently significant to result in a detectable interventricular mechanical delay in the LBBP group. Interestingly, this meta-analysis found that the paced QRS duration was significantly shorter in the LBBP group than the LVSP group. This suggests that although delayed RV excitation and interventricular dyssynchrony in the LBBP group might have been expected to prolong the paced QRS, the faster LVAT compensated for this, ultimately resulting in a shorter paced QRS duration.

### Cardiac function, structural remodeling, and clinical outcomes in patients with reduced LVEF and wide QRS duration

Observational studies demonstrated that a greater reduction in intrinsic QRS duration after CRT implantation was associated with greater improvements in LV systolic function and structural reverse remodeling.^52^^,^^53^ Lapidot et al^54^ revealed that acute narrowing of QRS duration after CRT implantation independently reduced the risk of all-cause mortality or HFH. Consistent with these findings, our meta-analysis demonstrated that the decrease in native QRS duration was significantly greater in the LBBP group, with an average reduction of approximately 21 ms than, the LVSP group in patients with a native QRS duration of >150 ms. Thus, LBBP exhibited superior efficacy compared with LVSP in enhancing both mechanical and electrophysiological remodeling of myocardial cells through various mechanisms, such as ion channel remodeling, improved β-adrenergic receptor responsiveness, and the reversal of gene expression dysregulation.^55^ These combined effects ultimately contribute to significant improvements in both cardiac function and structural remodeling. Moreover, this results in reductions in HFH and all-cause mortality.^55^ Consistent with this, this meta-analysis showed that the LBBP group had a 6% greater LVEF improvement than the LVSP group after a mean follow-up of 17 months. In addition, LVEDD decreased by 7 mm in the LBBP group and 3.5 mm in the LVSP group after a mean follow-up of 19 months in patients with large LVEDD, although the difference was not statistically significant. The risk of mortality and/or HFH was also significantly reduced in the LBBP group. Moreover, the ventricular pacing burden was similar between the groups, suggesting that it did not contribute to clinical outcomes.

The higher risk of mortality and HFH observed in the LVSP group may be attributable to several factors, including male sex, severe septal myocardial fibrosis, advanced structural heart disease, and a high incidence of ventricular arrhythmias and AF.^29^ However, multivariate analyses by Diaz et al^25^ and Zhu et al^29^ accounted for potential confounders such as age, sex, comorbidities, LVEF, LVEDD, N-terminal pro-brain natriuretic peptide levels, and heart failure medications. Furthermore, a pooled analysis from both studies showed that LBBP significantly reduced the risk of all-cause mortality and/or HFH by 72% compared with LVSP in patients with reduced LVEF and wide QRS.

Ultimately, LBBP is preferred over LVSP in patients with reduced LVEF and wide QRS duration, because it demonstrates greater efficacy by significantly improving LVEF and reducing LVEDD, thereby lowering the risk of all-cause mortality and HFH compared with the LVSP group.

### Cardiac function, structural remodeling, and clinical outcomes in patients with preserved LVEF and narrow QRS duration

In patients with baseline narrow QRS duration, preserving interventricular synchrony is essential to reduce the future risk of PICM and HFH.^56^ This meta-analysis demonstrated that LBBP did not significantly increase QRS duration in patients with a baseline narrow QRS duration. In contrast, LVSP was associated with a significant prolongation of QRS duration by approximately 14 ms in these patients. However, the impact of this prolonged QRS duration seems minimal in terms of inducing LV systolic dysfunction and LV dilatation during the follow-up period. This is supported by our findings, which showed no significant differences in LVEF, LVEDD, and HFH risk during the mean follow-up of 16.8 months between the 2 groups in patients with preserved LVEF and narrow QRS duration. These results suggest that LVSP is equally effective as LBBP in preserving LV function and structure, with a comparable risk of HFH in patients with preserved LVEF and narrow QRS duration. However, because PICM can develop several years after implantation, future observational studies with longer follow-ups are necessary to assess the long-term efficacy of LBBP vs LVSP.^57^

### Study limitations

This meta-analysis has several primary limitations. First, half of the included studies were retrospective cohort studies, which increases the potential for recall and selection bias. Second, patients’ baseline characteristics (eg, age, race, and comorbidities), ventricular pacing burden, device programming, and the presence of coexisting arrhythmia may serve as confounding factors influencing the outcomes. Lastly, substantial heterogeneity was observed in several analyses, attributable to variations in the diagnostic criteria for LBBP and LVSP across studies and differences in baseline QRS duration, LVEF, and LVEDD. However, heterogeneity was significantly reduced through subgroup and sensitivity analyses, with the findings remaining robust and statistically significant.

Given the limited number of studies included in this meta-analysis that assess echocardiographic and clinical outcomes, additional observational studies are necessary to further validate these findings. Furthermore, additional observational studies are required to facilitate a more precise comparison of efficacy and safety in future meta-analyses aimed at identifying the optimal pacing site that maximizes efficacy while minimizing procedural complications. These studies should (1) use standardized diagnostic criteria for LBBP and LVSP, as recommended in recent guidelines^11^; (2) compare echocardiographic and clinical outcomes between s-LBBP and ns-LBBP; and (3) evaluate the efficacy and safety of different LBBP classifications, including left bundle trunk pacing vs left bundle fascicular pacing.^31^^,^^58^

## Conclusion

In patients with reduced LVEF and wide QRS duration, LBBP demonstrated superior efficacy over LVSP in maintaining intraventricular synchrony, which resulted in improved LV systolic function and remodeling, ultimately lowering the risk of mortality and HFH. In contrast, in patients with preserved LVEF and narrow QRS duration, LVSP was noninferior to LBBP in preserving LV function and structure, with a similar HFH risk. Finally, both groups exhibited comparable safety profiles and procedural efficiency.
