# Supplementary material for: Efficacy and safety of left bundle branch pacing compared with left ventricular septal pacing: A systematic review and meta-analysis
Source: Heart Rhythm O2. 2025 Aug 13;6(9):1335–55. doi: 10.1016/j.hroo.2025.06.006 (PMC12635747; doi:10.1016/j.hroo.2025.06.006)
Supplement: Supplementary Table and Figures [file mmc1.docx]

**Supplementary Table S1.** Newcastle-Ottawa Scale for Included Studies

| **Authors (Year)** | Exposed truly representative | Selection of non-exposed from the same community | Exposure ascertained by secure record or interview | Demonstration of outcome of interest not present at the start of the study | Study controls for other variables | Assessment of outcome | Follow up long enough for outcome to occur | Complete follow up of all subjects accounted | Subject lost to follow up unlikely to introduce bias | Score | Risk of bias |
| --- | --- | --- | --- | --- | --- | --- | --- | --- | --- | --- | --- |
| Qian 2021 | 1 | 1 | 1 | 1 | 0 | 1 | 0 | 1 | 1 | 7 | Low |
| Zhang 2021 | 1 | 1 | 1 | 1 | 0 | 1 | 1 | 1 | 1 | 8 | Low |
| Curila 2021 | 1 | 1 | 1 | 1 | 0 | 1 | 0 | 1 | 1 | 7 | Low |
| Wu 2021 | 1 | 1 | 1 | 1 | 0 | 1 | 0 | 1 | 1 | 7 | Low |
| Shimeno 2021 | 1 | 1 | 1 | 1 | 0 | 1 | 0 | 1 | 1 | 7 | Low |
| Heckman 2021 | 1 | 1 | 1 | 1 | 0 | 1 | 0 | 1 | 1 | 7 | Low |
| Jastrzebski 2022 | 1 | 1 | 1 | 1 | 0 | 1 | 1 | 1 | 1 | 8 | Low |
| Jastrzebski 2022 | 1 | 1 | 1 | 1 | 0 | 1 | 0 | 1 | 1 | 7 | Low |
| Zhou 2022 | 1 | 1 | 1 | 1 | 0 | 1 | 1 | 1 | 1 | 8 | Low |
| Shimeno 2022 | 1 | 1 | 1 | 1 | 0 | 1 | 0 | 1 | 1 | 7 | Low |
| Qian 2022 | 1 | 1 | 1 | 1 | 0 | 1 | 0 | 1 | 1 | 7 | Low |
| Zhang 2023 | 1 | 1 | 1 | 1 | 0 | 1 | 1 | 1 | 1 | 8 | Low |
| Kato 2023 | 1 | 1 | 1 | 1 | 0 | 1 | 1 | 1 | 1 | 8 | Low |
| Peng 2023 | 1 | 1 | 1 | 1 | 0 | 1 | 1 | 1 | 1 | 8 | Low |
| Shen 2023 | 1 | 1 | 1 | 1 | 0 | 1 | 0 | 1 | 1 | 7 | Low |
| Shen 2024 | 1 | 1 | 1 | 1 | 0 | 1 | 0 | 1 | 1 | 7 | Low |
| Cheng 2024 | 1 | 1 | 1 | 1 | 0 | 1 | 1 | 1 | 1 | 8 | Low |
| Diaz 2024 | 1 | 1 | 1 | 1 | 1 | 1 | 1 | 1 | 1 | 9 | Low |
| Cano 2024 | 1 | 1 | 1 | 1 | 0 | 1 | 1 | 1 | 1 | 8 | Low |
| Rijks 2024 | 1 | 1 | 1 | 1 | 0 | 1 | 0 | 1 | 1 | 7 | Low |
| Zhu 2024 | 1 | 1 | 1 | 1 | 1 | 1 | 1 | 1 | 1 | 9 | Low |
| Chen 2024 | 1 | 1 | 1 | 1 | 0 | 1 | 1 | 1 | 1 | 8 | Low |

|  |
| --- |
|  |


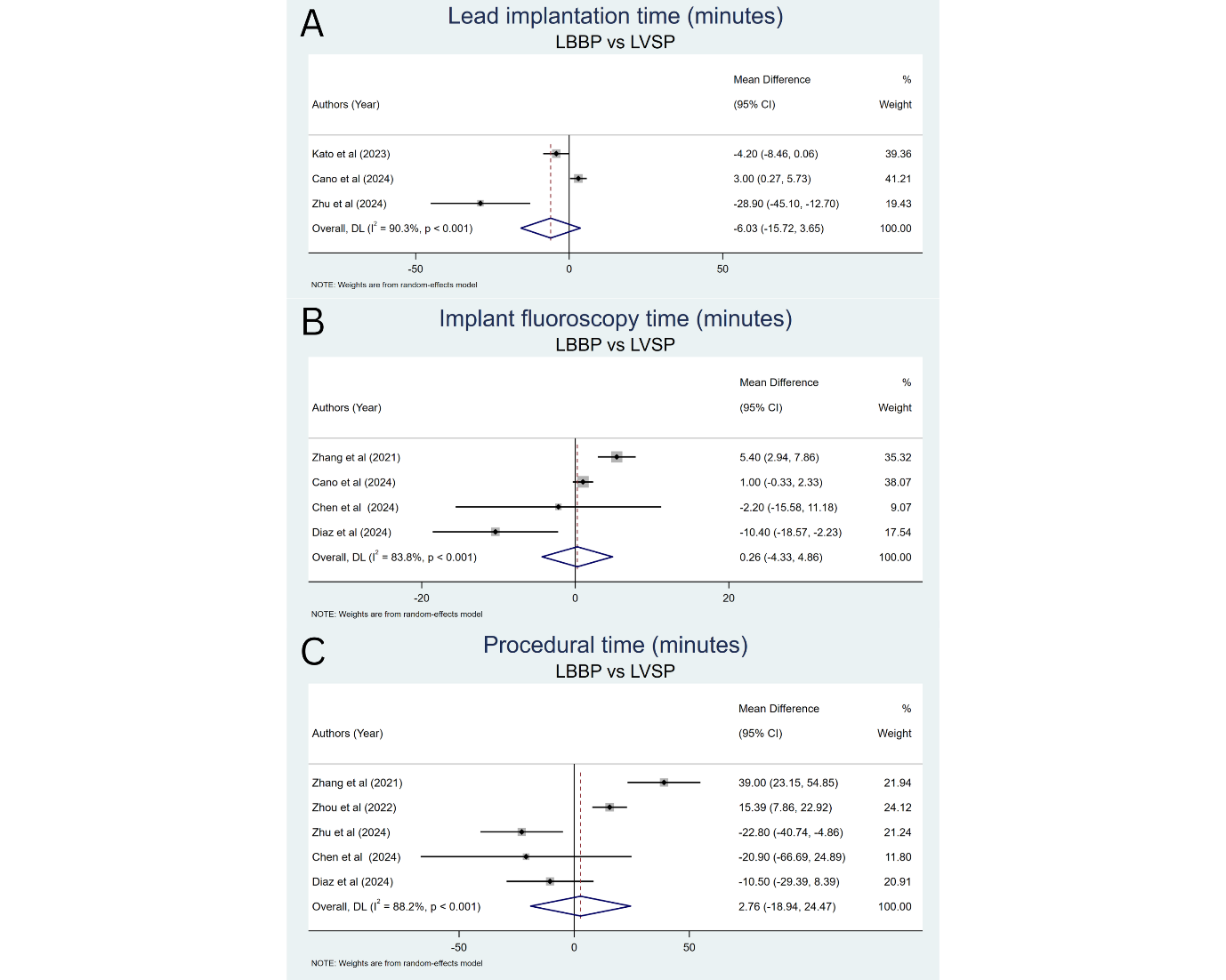


**Supplementary Figure S1.** Comparison of procedural duration between LBBP and LVSP.

1. Lead implantation time (minutes); (B) Implant fluoroscopy time (minutes); (C) Procedural time (minutes).

LBBP: left bundle branch pacing; LVSP: left ventricular septal pacing.


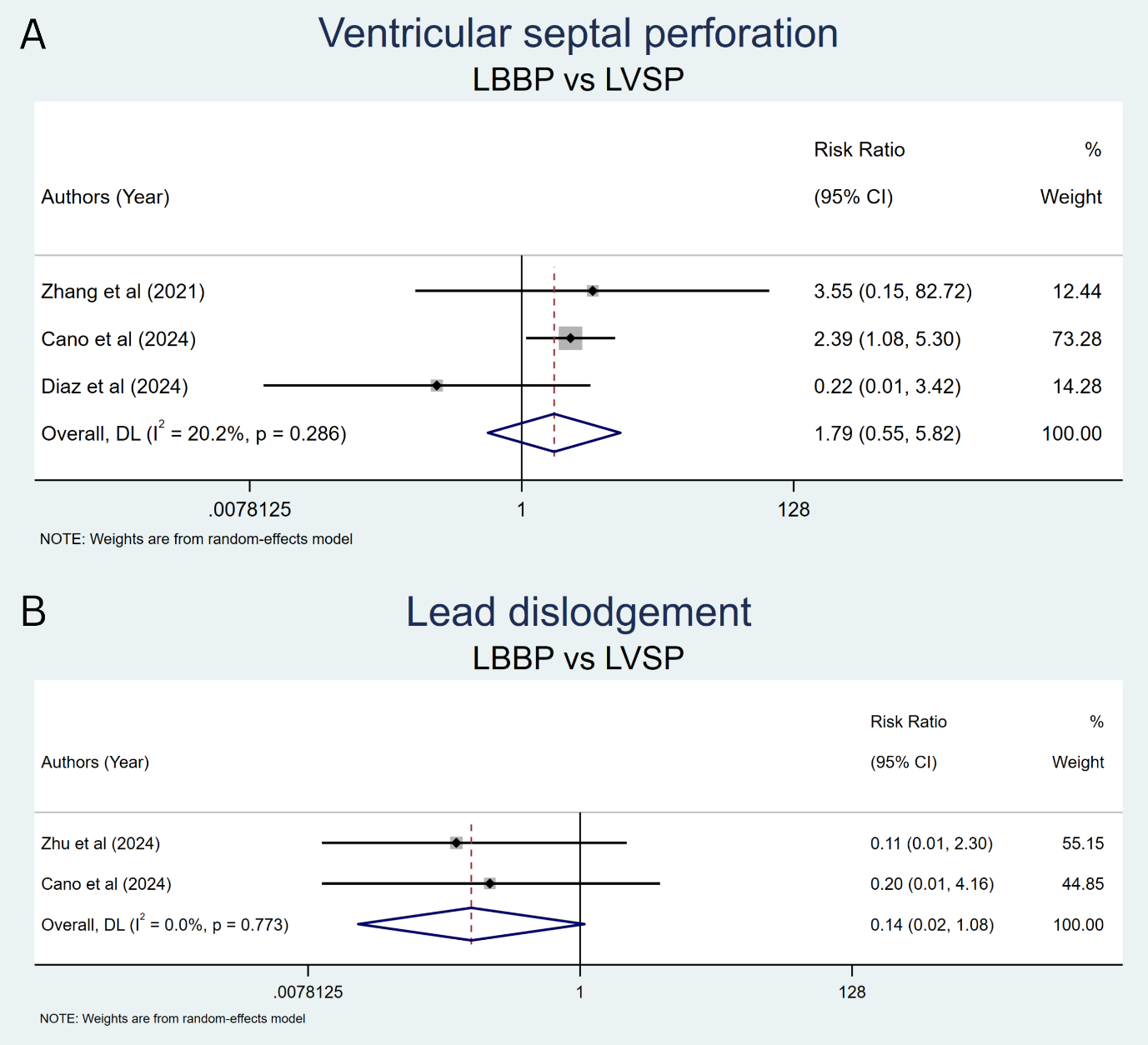


**Supplementary Figure S2.** Comparison of procedural complications between LBBP and LVSP.

1. Ventricular septal perforation (B) Lead dislodgement.

LBBP: left bundle branch pacing; LVSP: left ventricular septal pacing.


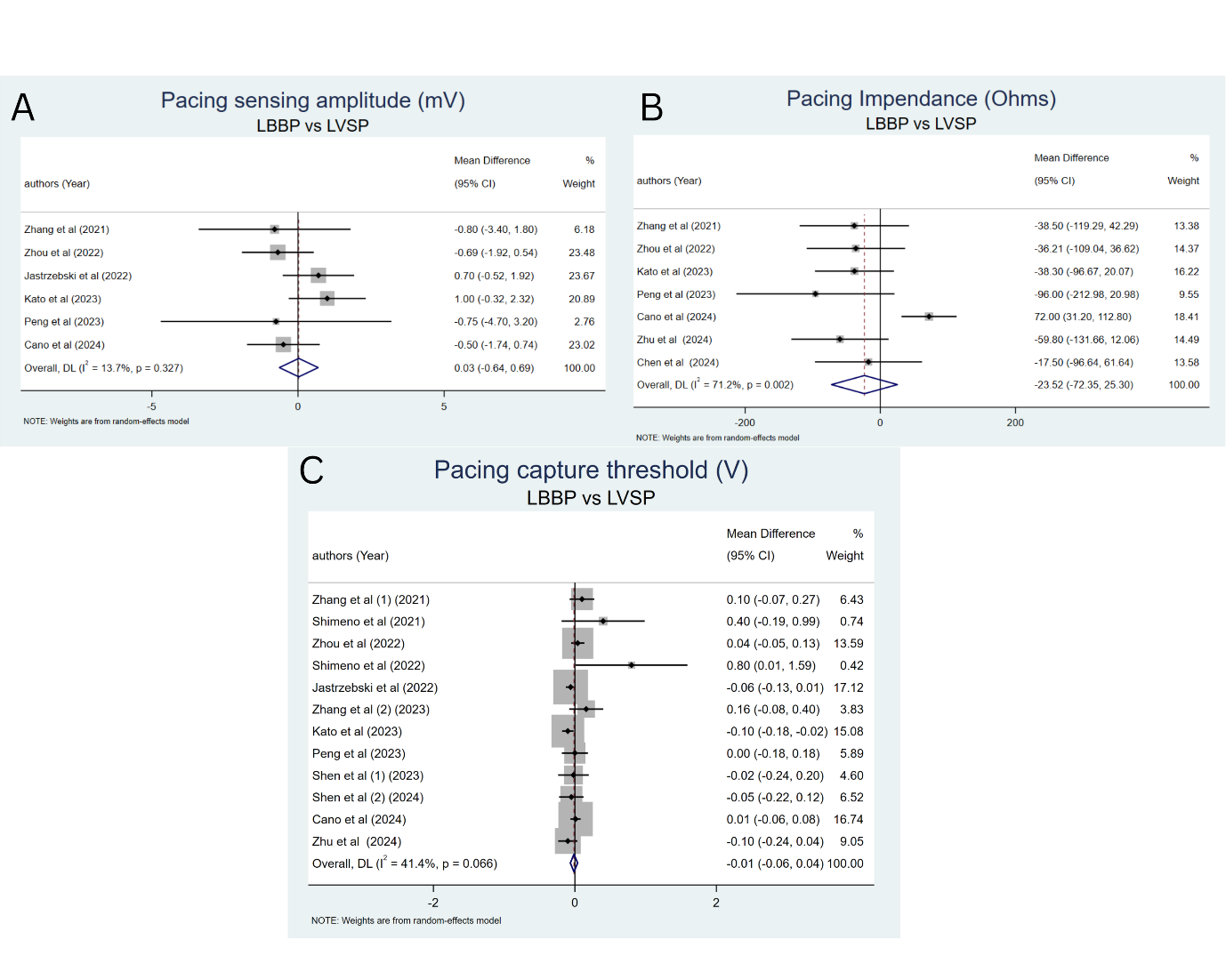


**Supplementary Figure S3.** Comparison of pacing parameters postoperatively between LBBP and LVSP.

1. Pacing sensing amplitude (milli Volt); (B) Pacing impedance (Ohms); (C) Pacing capture threshold (Volt).

LBBP: left bundle branch pacing; LVSP: left ventricular septal pacing; mV: millivolt; V: volt.


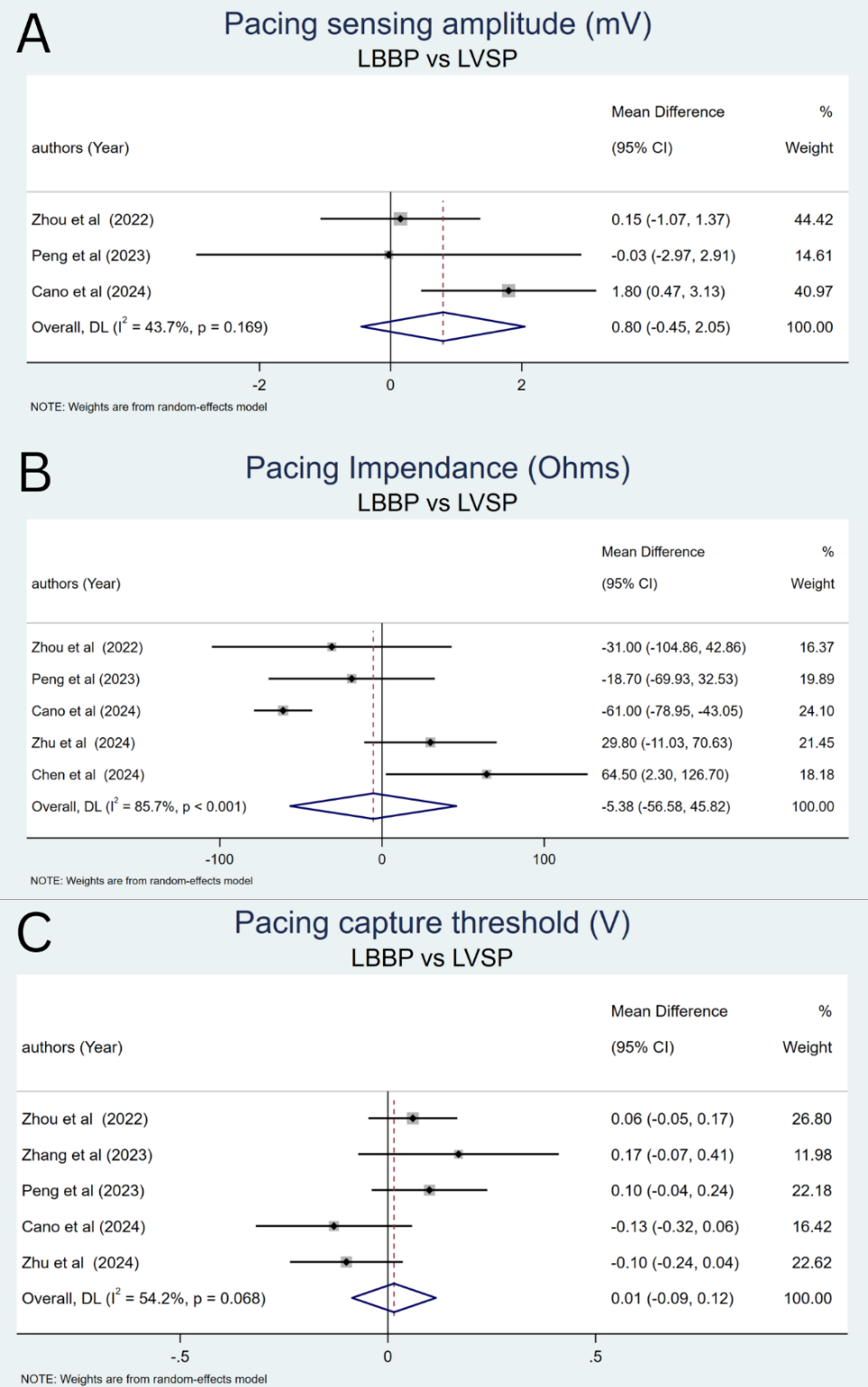


**Supplementary Figure S4.** Comparison of pacing parameters during follow-up between LBBP and LVSP.

1. Pacing sensing amplitude (milli Volt); (B) Pacing impedance (Ohms); (C) Pacing capture threshold (Volt).

LBBP: left bundle branch pacing; LVSP: left ventricular septal pacing; mV: millivolt; V: volt.


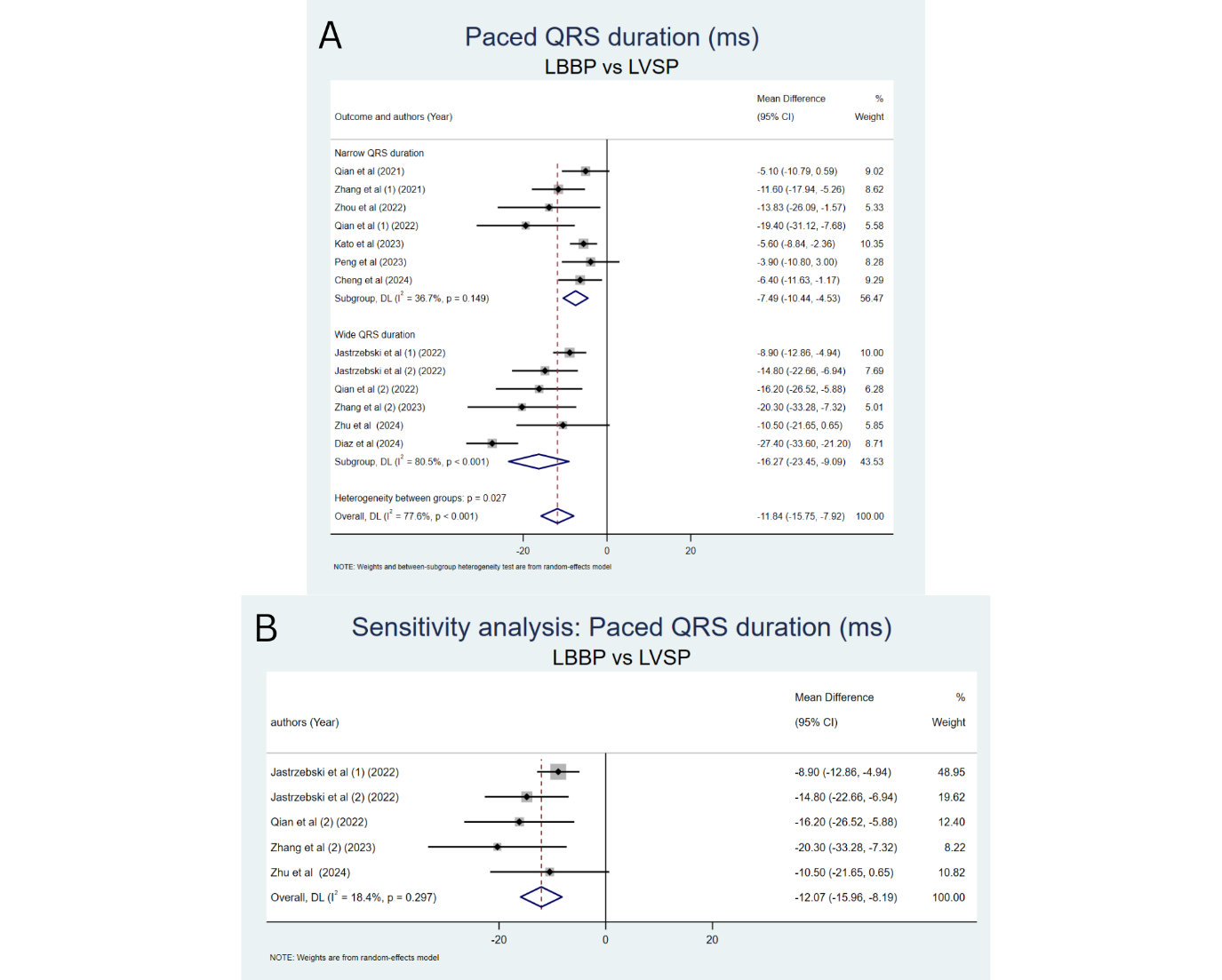


**Supplementary Figure S5.** Sub-group analysis and sensitivity analyses of paced QRS duration.

1. Sub-group analysis of paced QRS duration (ms); (B) Sensitivity analysis of paced QRS duration (ms).

ms: milliseconds; LBBP: left bundle branch pacing; LVSP: left ventricular septal pacing.

**
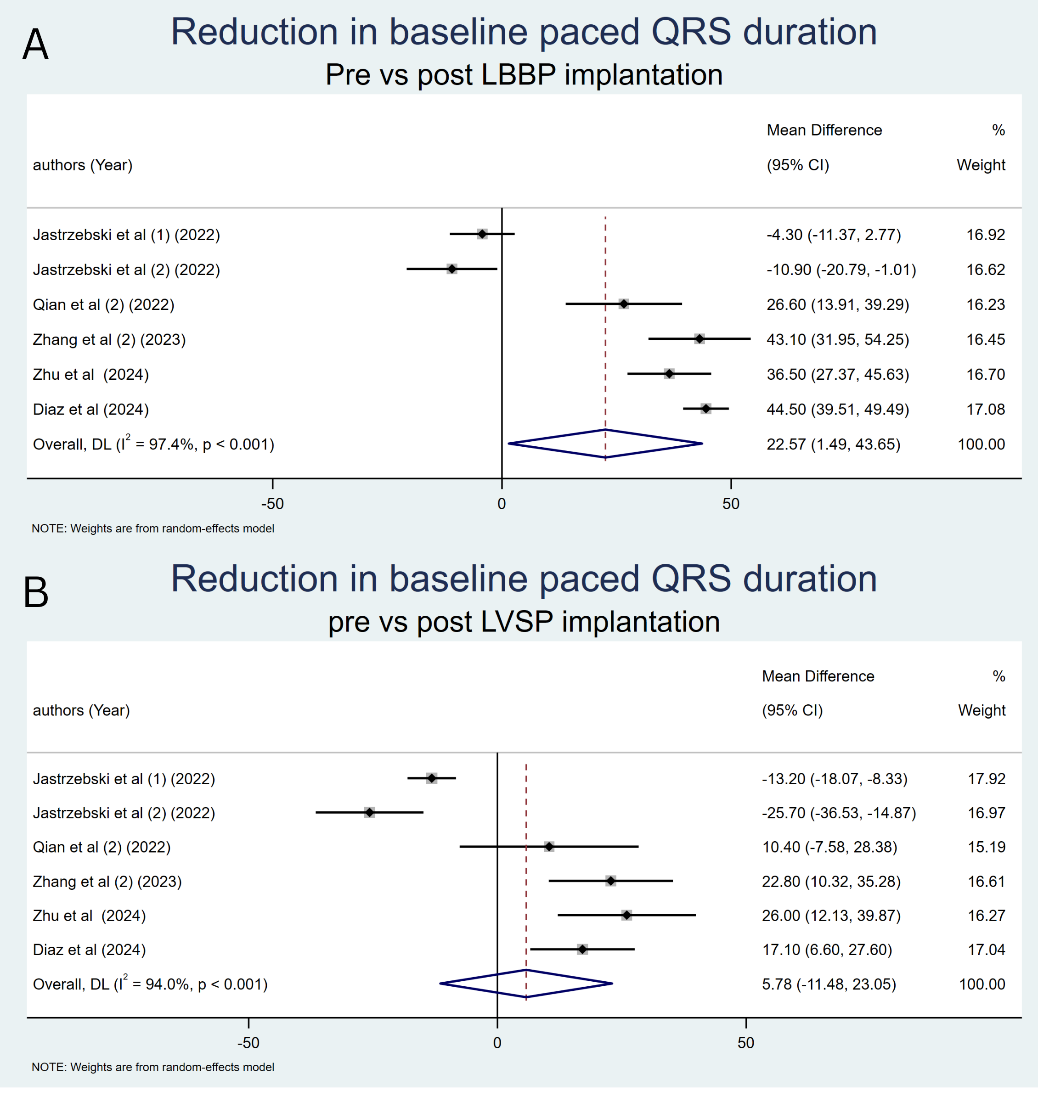
**

**Supplementary Figure S6.** Comparison of reduction in baseline paced QRS duration between LBBP and LVSP in patients with wide QRS duration.

1. Reduction in baseline paced QRS duration in LBBP group (ms); (B) Reduction in baseline paced QRS duration in LVSP group (ms).

ms: milliseconds; LBBP: left bundle branch pacing; LVSP: left ventricular septal pacing.


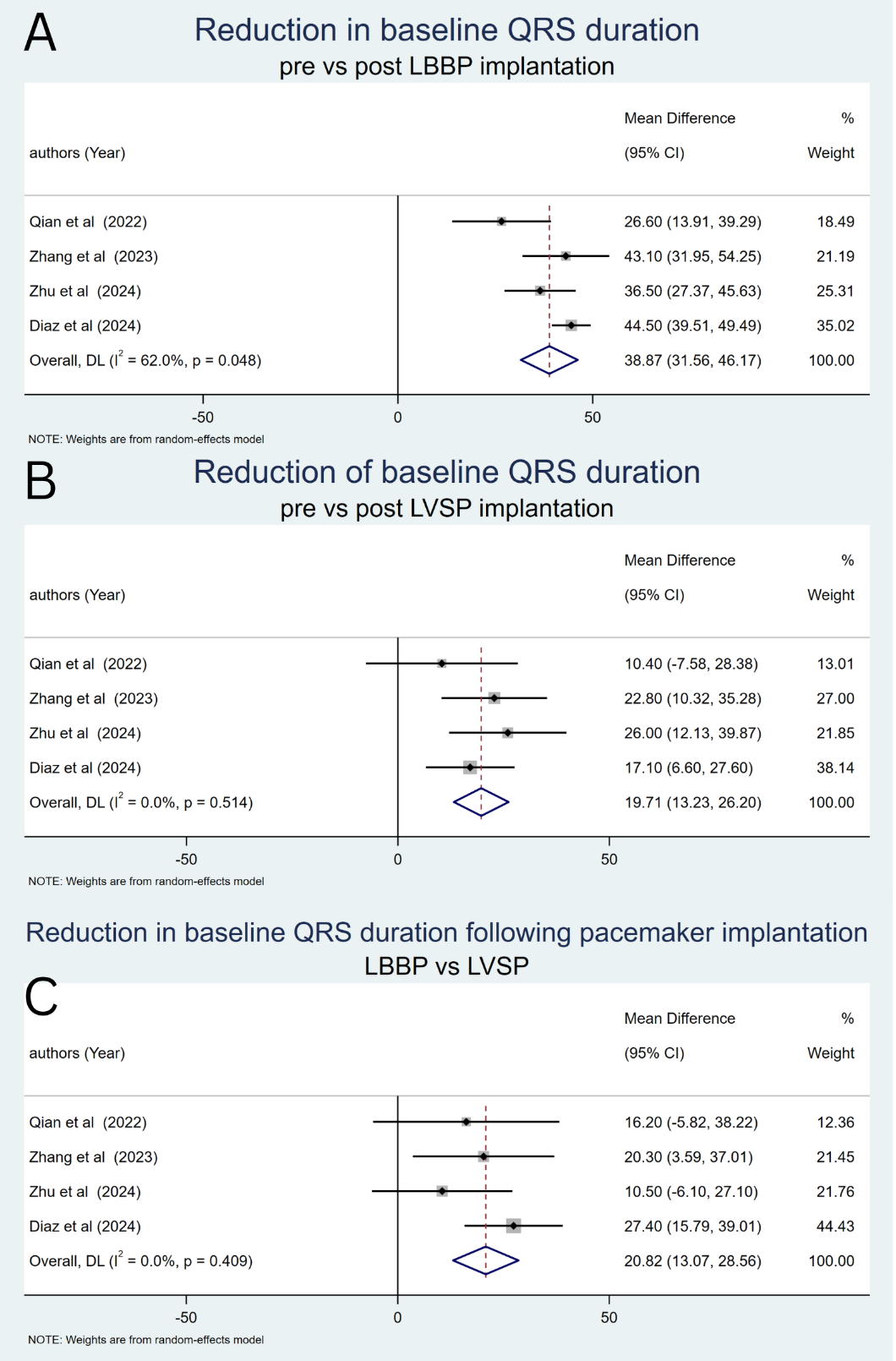


**Supplementary Figure S7.** Comparison of reduction in baseline paced QRS duration between LBBP and LVSP in patients with baseline QRS duration > 150 ms.

1. Reduction in baseline paced QRS duration in LBBP group (ms); (B) Reduction in baseline paced QRS duration in LVSP group (ms); (C) The difference of reduction in baseline paced QRS duration between LBBP and LVSP.

ms: milliseconds; LBBP: left bundle branch pacing; LVSP: left ventricular septal pacing.

**
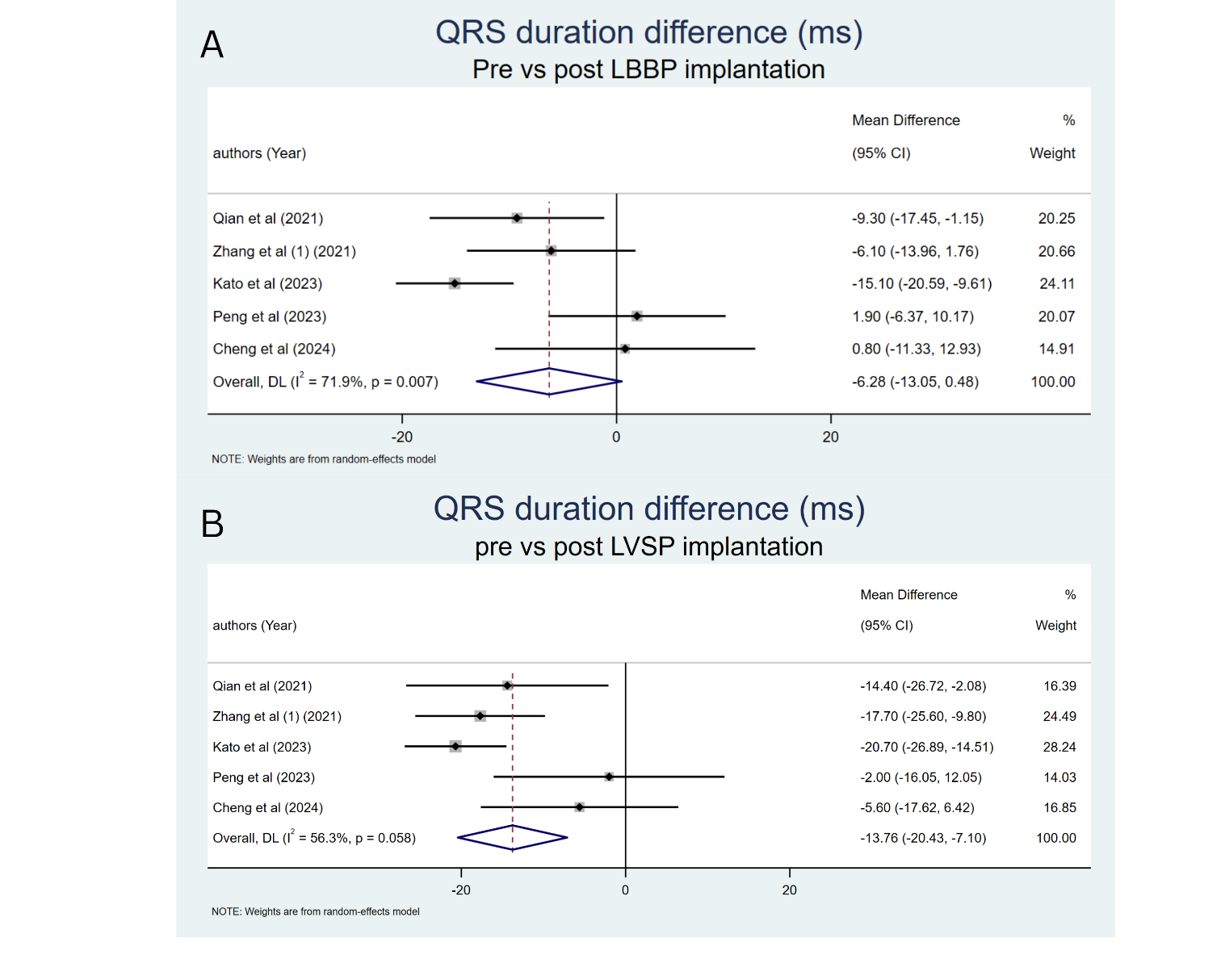
**

**Supplementary Figure S8.** Comparison of reduction in baseline QRS duration between LBBP and LVSP in patients with narrow QRS duration.

1. QRS duration difference in LBBP group (ms); (B) QRS duration difference in LVSP group (ms).

ms: milliseconds; LBBP: left bundle branch pacing; LVSP: left ventricular septal pacing.


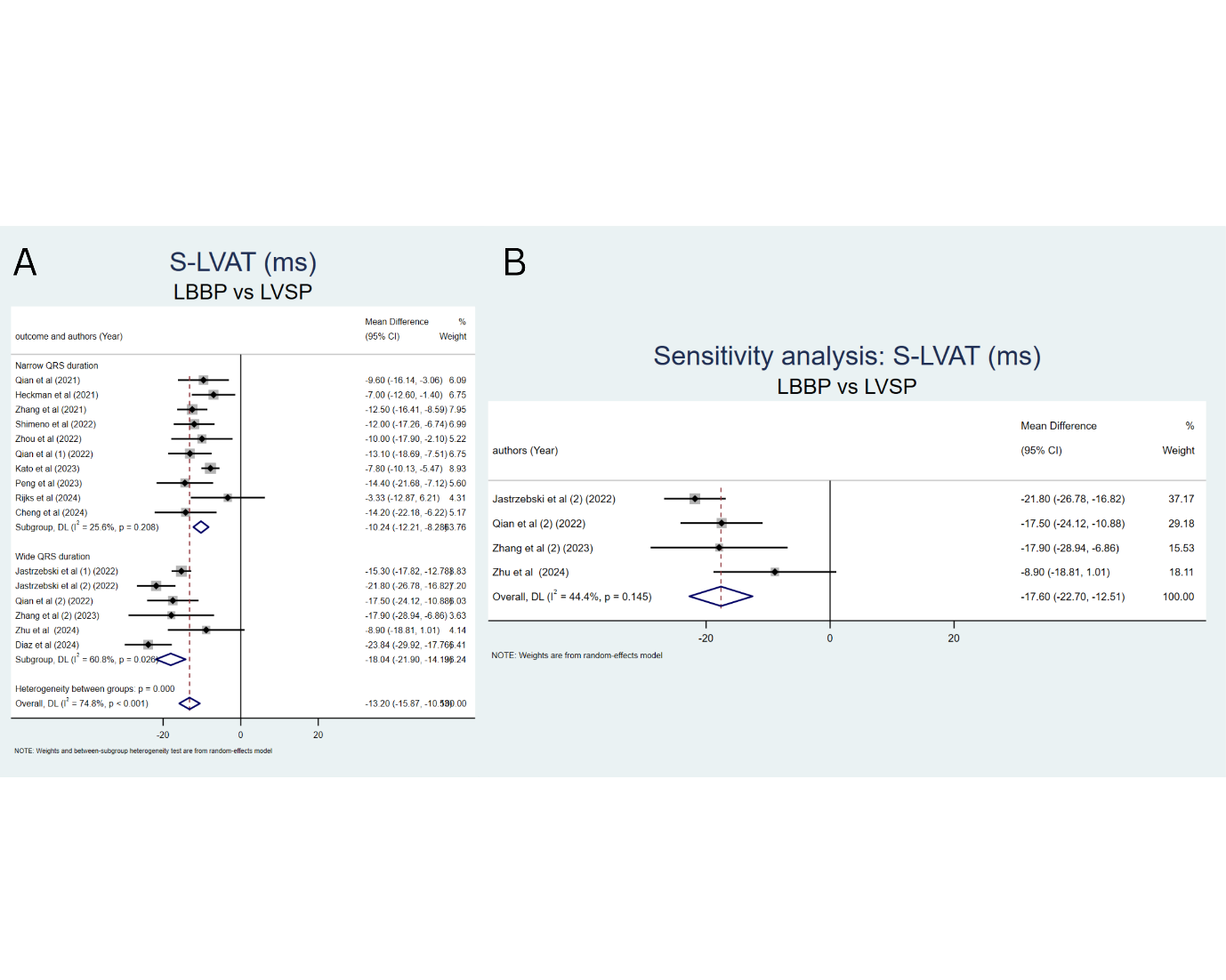


**Supplementary Figure S9.** Sub-group analysis and sensitivity analyses of S-LVAT between LBBP and LVSP groups.

1. Sub-group analysis of S-LVAT (ms); (B) Sensitivity analysis of S-LVAT (ms).

ms: milliseconds; LBBP: left bundle branch pacing; LVSP: left ventricular septal pacing; S-LVAT: stimulation to left ventricular activation time.


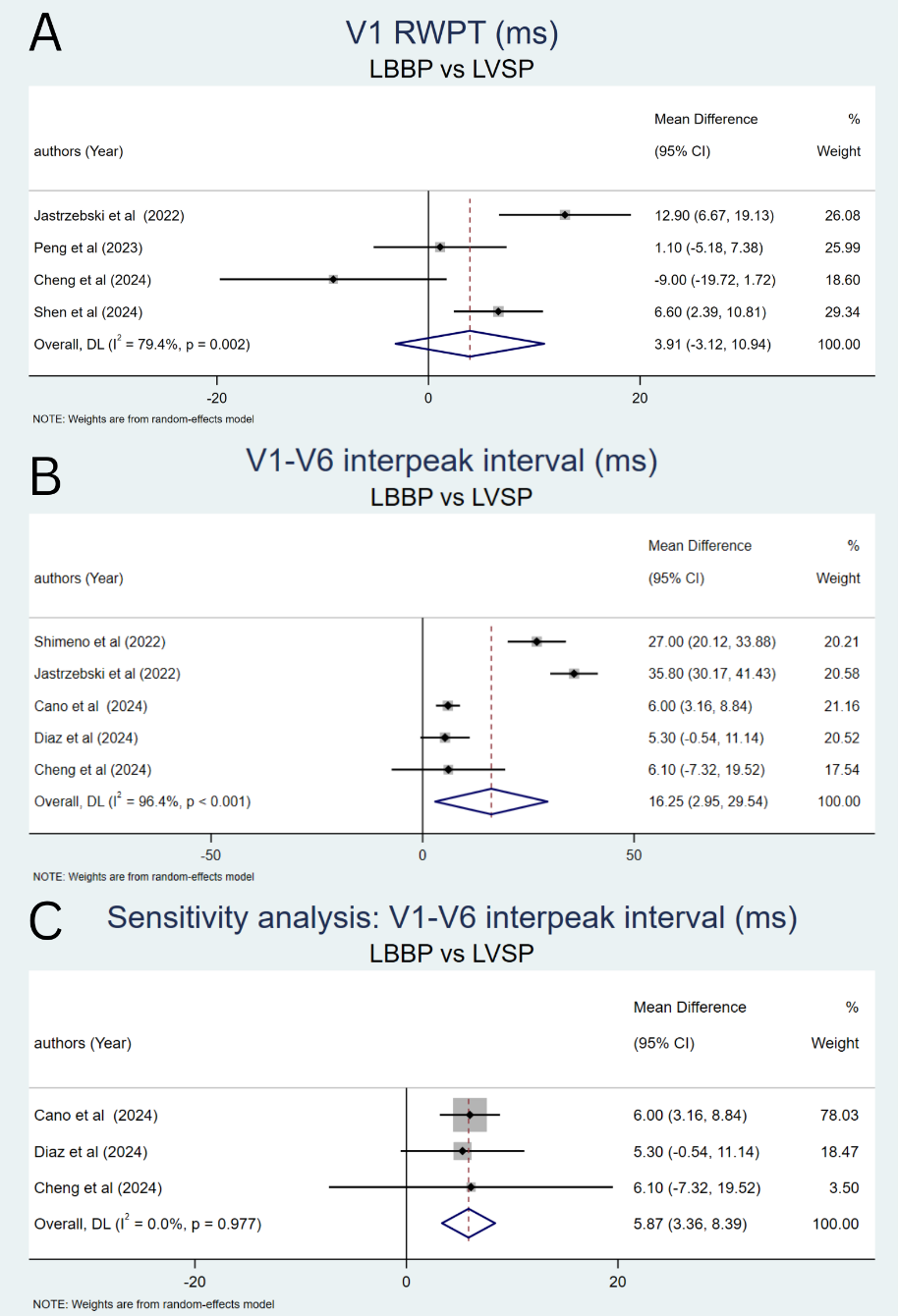


**Supplementary Figure S10.** Comparison of V1 RWPT and V1-V6 interpeak interval between LBBP and LVSP.

1. Comparison of V1 RWPT between LBBP and LVSP; (B) Comparison of V1-V6 interpeak interval between LBBP and LVSP; (C) Sensitivity analysis of V1-V6 interpeak interval between LBBP and LVSP

ms: milliseconds; LBBP: left bundle branch pacing; LVSP: left ventricular septal pacing; RWPT: R wave peak time.

**
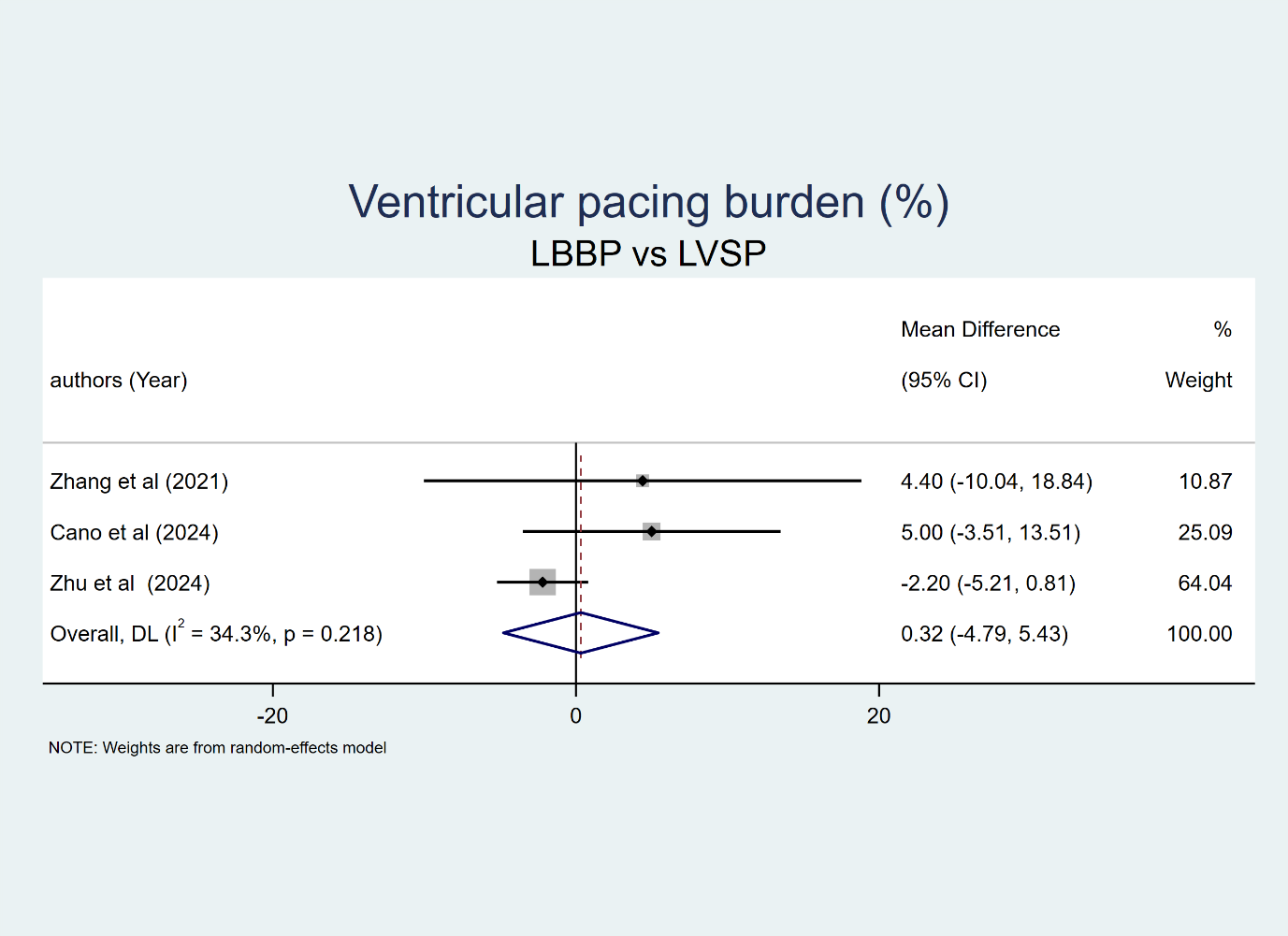
**

**Supplementary Figure S11.** Comparison of ventricular pacing burden between LBBP and LVSP.

LBBP: left bundle branch pacing; LVSP: left ventricular septal pacing.


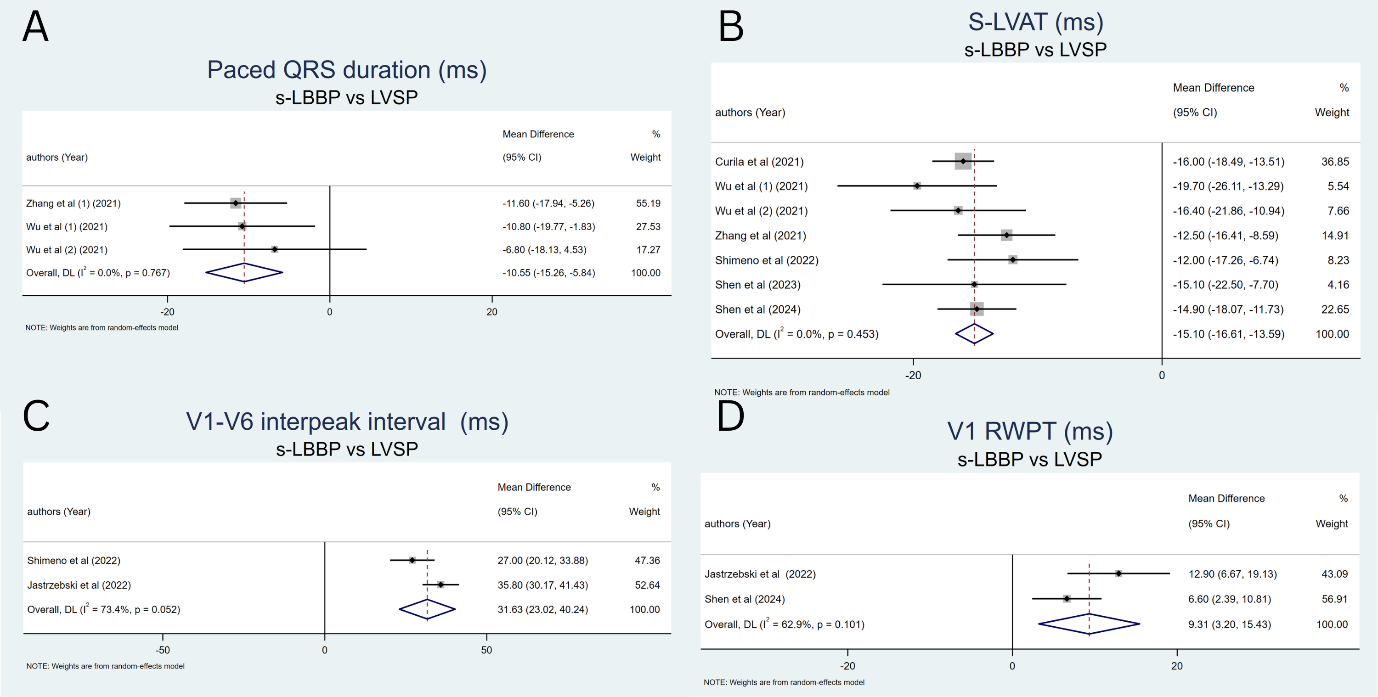


**Supplementary Figure S12.** Comparison of pacing parameters between S-LBBP and LVSP.

1. Paced QRS duration (ms); (B) S-LVAT (ms); (C) V1-V6 interpeak interval (ms); (D) V1 RWPT (ms)

ms: milliseconds; s-LBBP: spesific left bundle branch pacing; LVSP: left ventricular septal pacing; RWPT: R wave peak time


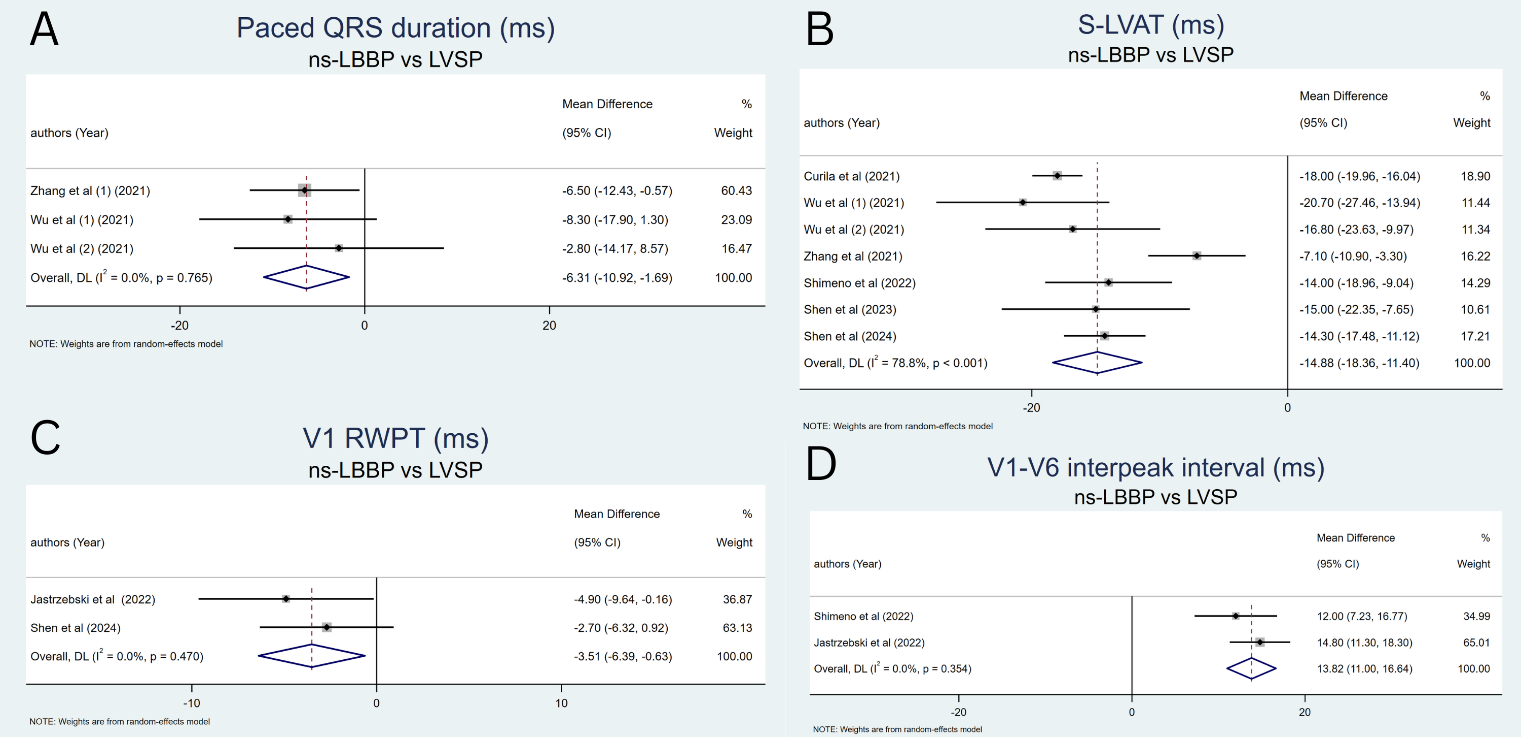


**Supplementary Figure S13.** Comparison of pacing parameters between ns-LBBP and LVSP.

1. Paced QRS duration (ms); (B) S-LVAT (ms); (C) V1 RWPT (ms); (D) V1-V6 interpeak interval (ms).

ms: milliseconds; ns-LBBP: non-spesific left bundle branch pacing; LVSP: left ventricular septal pacing; RWPT: R wave peak time


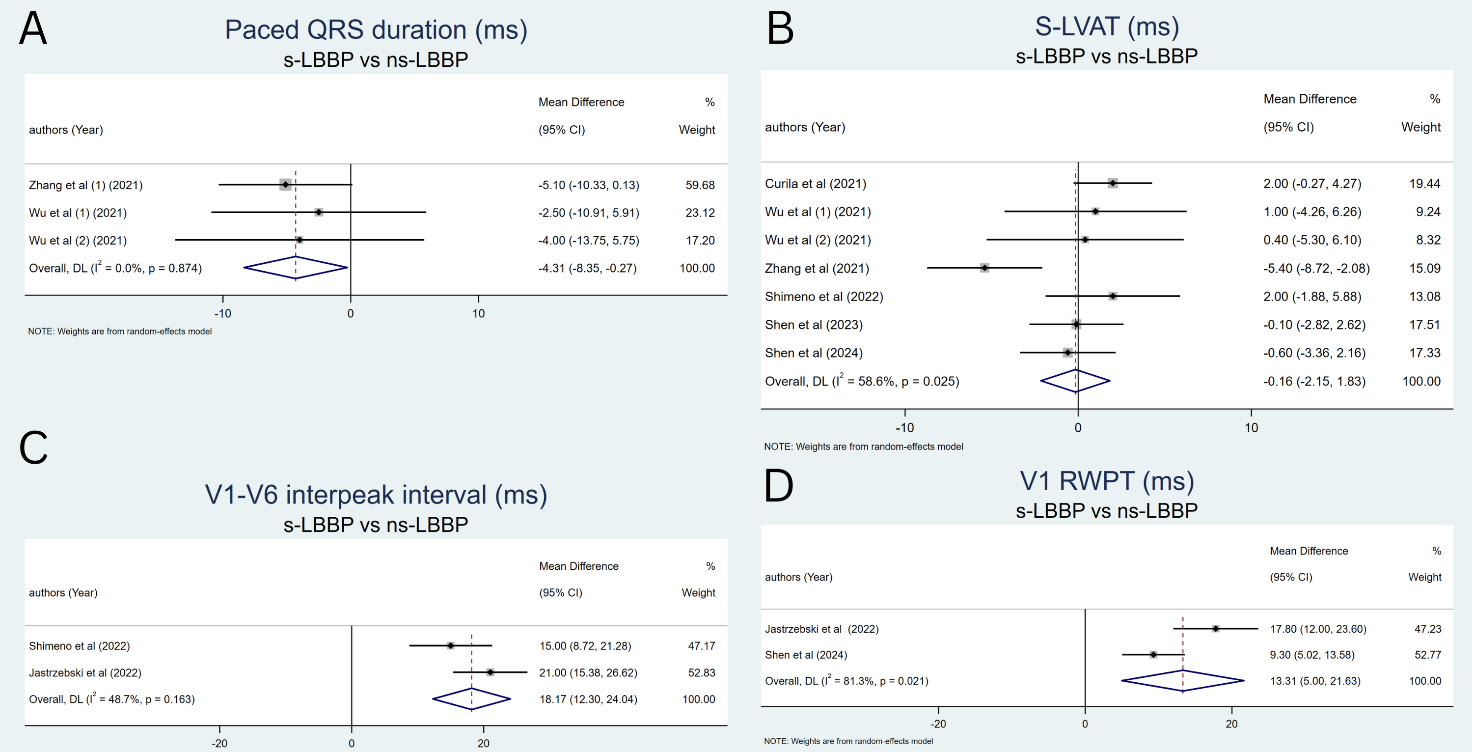


**Supplementary Figure S14.** Comparison of pacing parameters between s-LBBP and ns-LBBP.

1. Paced QRS duration (ms); (B) S-LVAT (ms); (C) V1-V6 interpeak interval (ms); (D) V1 RWPT (ms).

ms: milliseconds; s-LBBP: spesific left bundle branch pacing; ns-LBBP: non-spesific left bundle branch pacing; LVSP: left ventricular septal pacing; RWPT: R wave peak time.


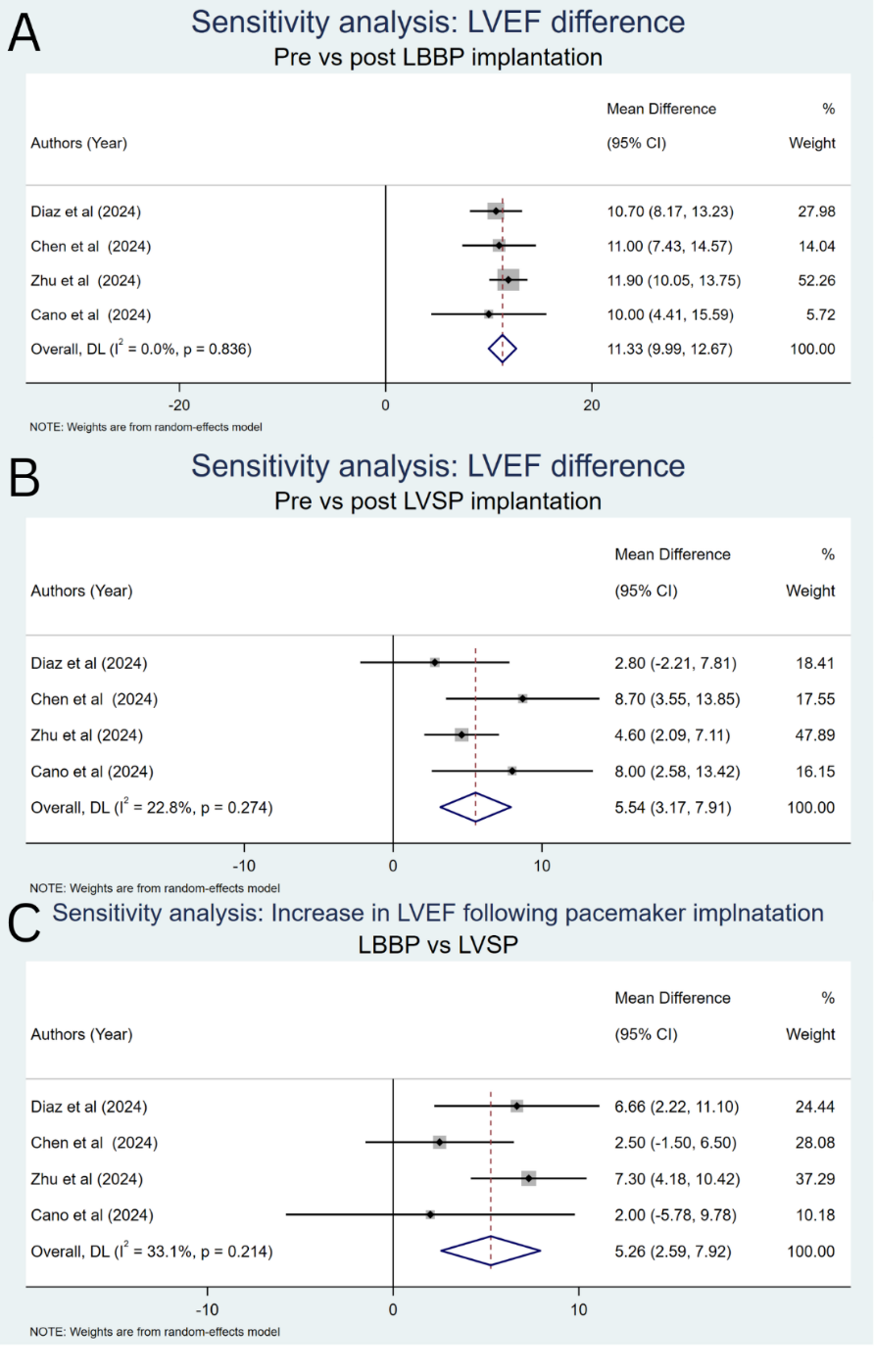


**Supplementary Figure S15.** Sensitivity analysis of LVEF outcomes between LBBP and LVSP.

1. LVEF difference in LBBP group; (B) LVEF difference in LVSP group; (C) comparison of increase in LVEF between LBBP and LVSP groups.

LBBP: left bundle branch pacing; LVSP: left ventricular septal pacing; LVEF: left ventricular ejection fraction.


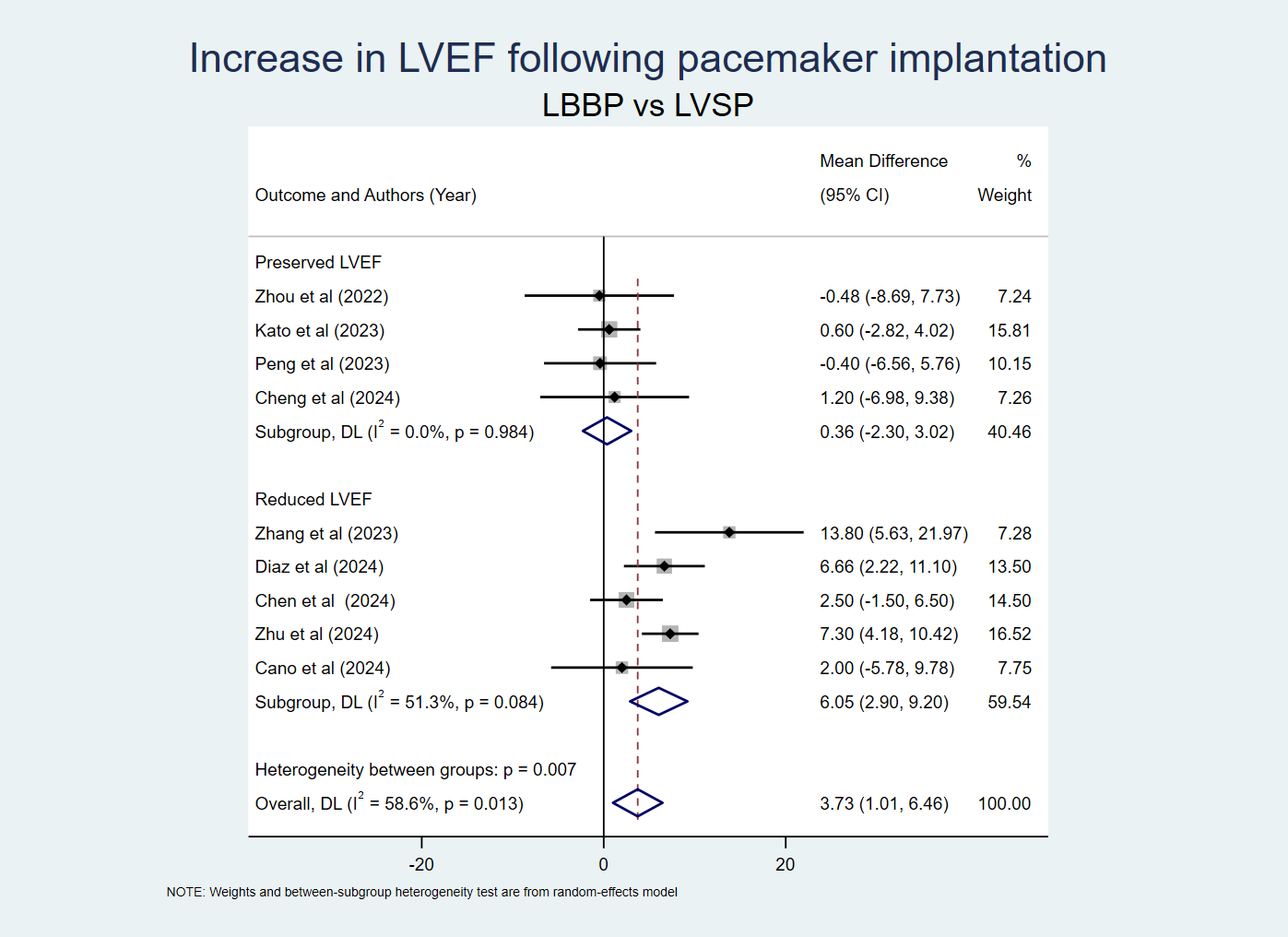


**Supplementary Figure S16.** Comparison of increase in LVEF between LBBP and LVSP.

LBBP: left bundle branch pacing; LVSP: left ventricular septal pacing: LVEF: left ventricular ejection fraction.


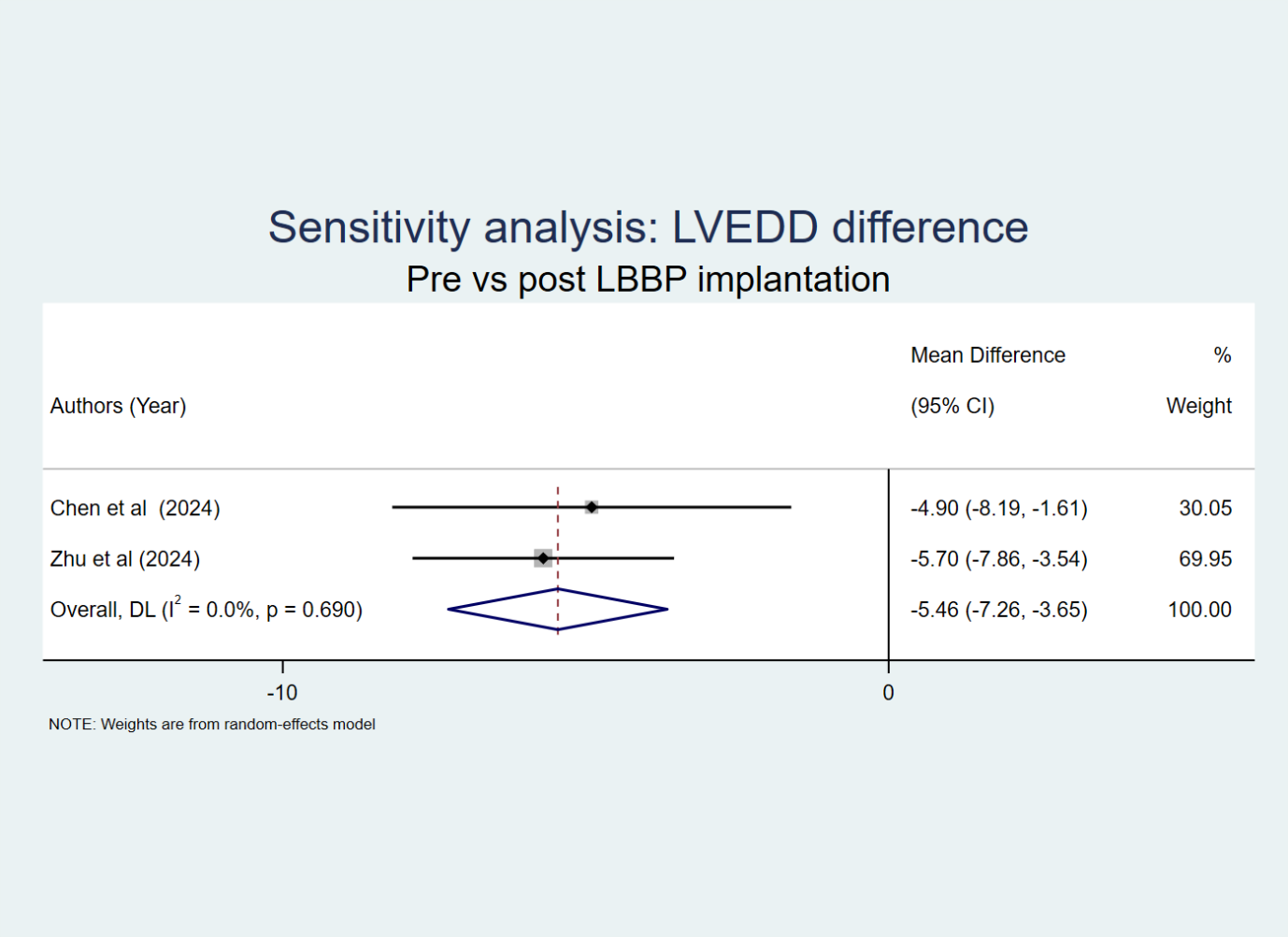


**Supplementary Figure S17.** Sensitivity analysis of LVEDD difference in LBBP group.

LBBP: left bundle branch pacing; LVSP: left ventricular septal pacing; LVEDD: left ventricular end diastolic diameter.


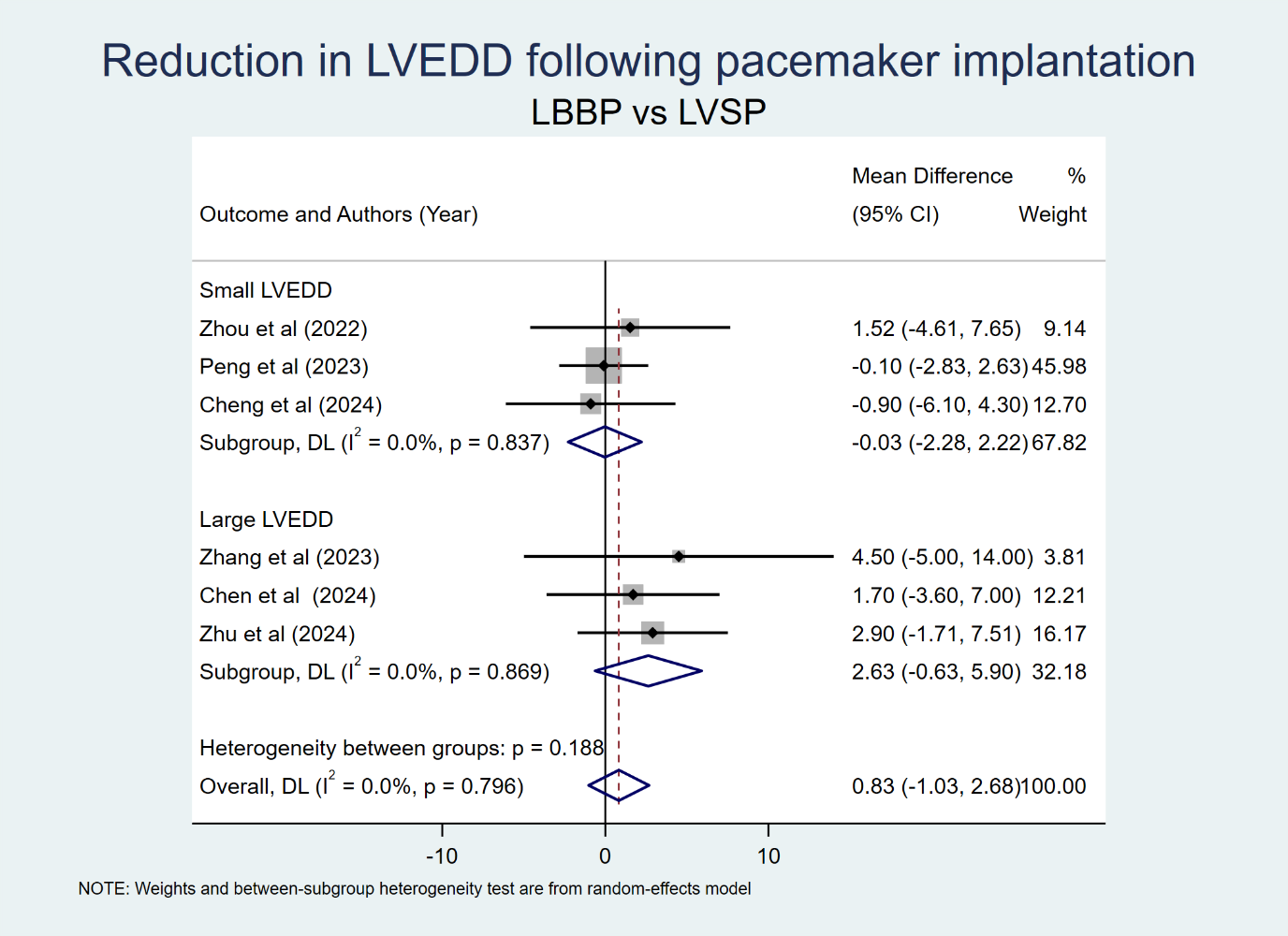


**Supplementary Figure S18.** Comparison of reduction in LVEDD between LBBP and LVSP.

LBBP: left bundle branch pacing; LVSP: left ventricular septal pacing; LVEDD: left ventricular end diastolic diameter.


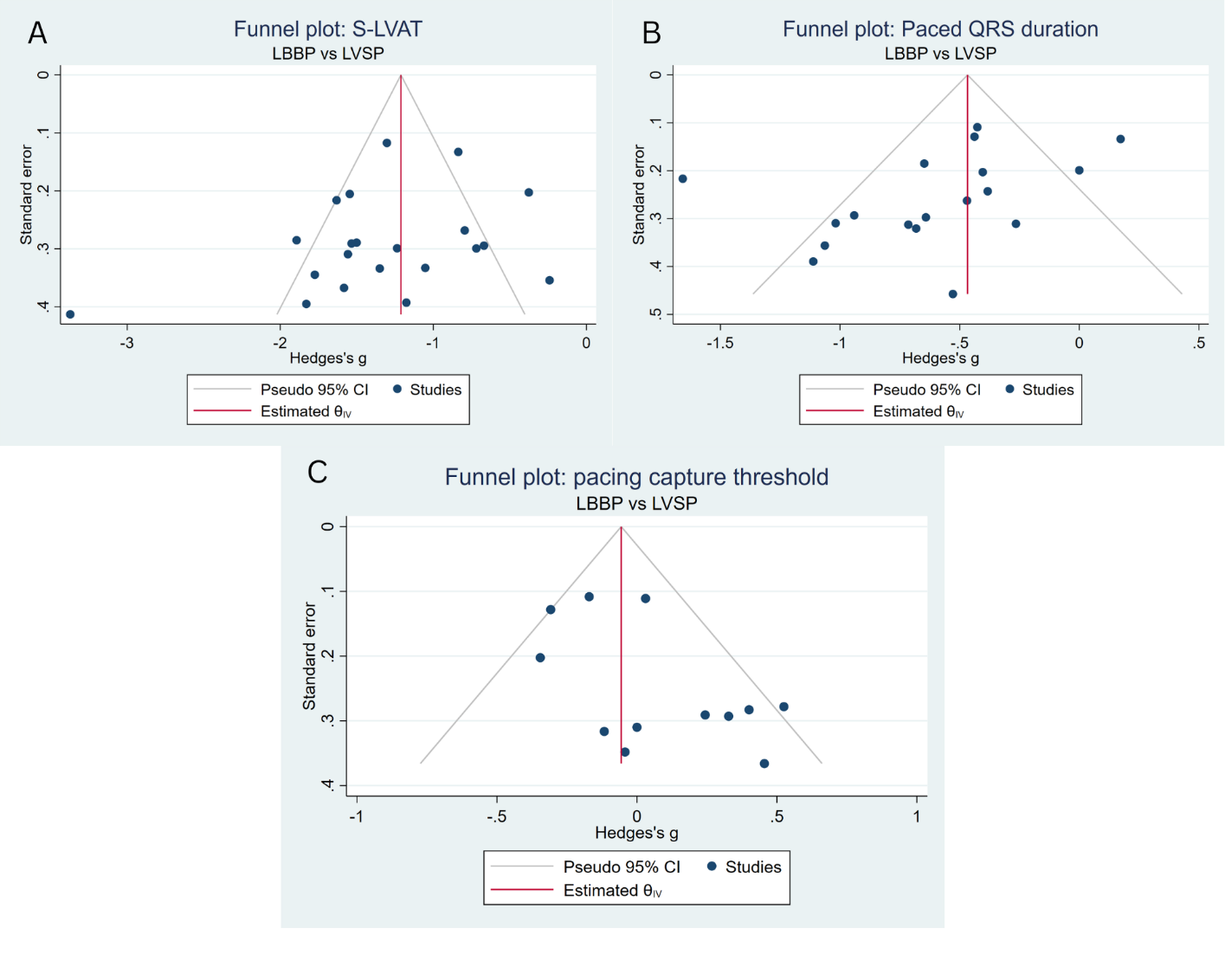


**Supplementary Figure S19.** Begg’s funnel plot of S-LVAT, paced QRS duration, and pacing capture threshold outcomes.

1. S-LVAT; (B) Paced QRS duration; (C) Pacing capture threshold.

LBBP: left bundle branch pacing; LVSP: left ventricular septal pacing.
